# Supplementary material for: Matrin3 mediates differentiation through stabilizing chromatin loop-domain interactions and YY1 mediated enhancer-promoter interactions
Source: Nat Commun. 2024 Feb 10;15:1274. doi: 10.1038/s41467-024-45386-w (PMC10858947; doi:10.1038/s41467-024-45386-w)
Supplement: Supplementary file 1 — Supplementary Information [file 41467_2024_45386_MOESM1_ESM.pdf]

Supplementary Information

Matrin3 mediates differentiation through stabilizing chromatin loop-domain interactions and YY1 mediated enhancer-promoter interactions

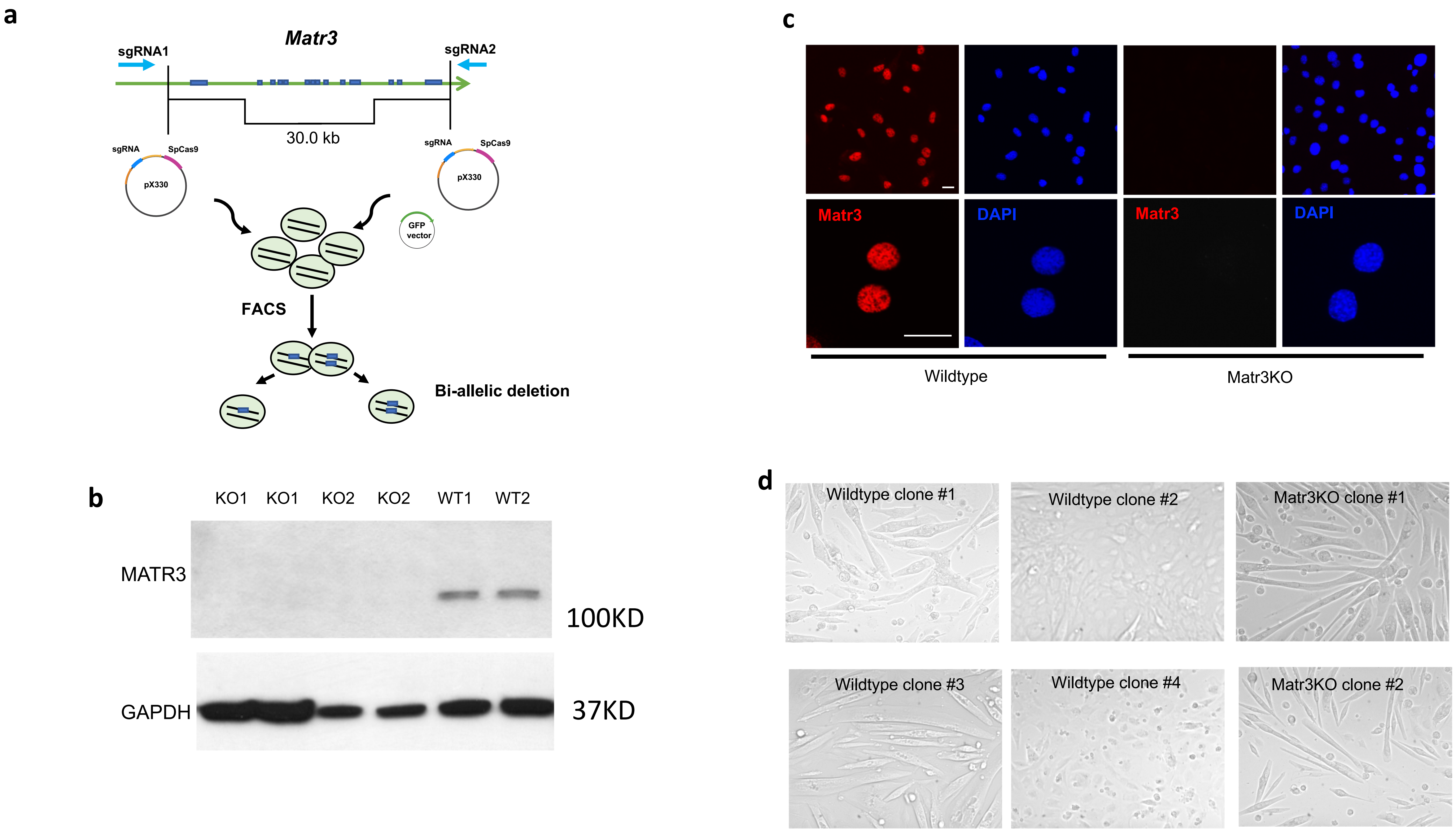
a
Matr3
sgRNA1
sgRNA2
30.0 kb
sgRNA
SpCas9
pX330
sgRNA
SpCas9
pX330
GFP vector
FACS
Bi-allelic deletion
b
KO1
KO1
KO2
KO2
WT1
WT2
MATR3
100KD
GAPDH
37KD
c
Matr3
DAPI
Matr3
DAPI
Wildtype
Matr3KO
d
Wildtype clone #1
Wildtype clone #2
Matr3KO clone #1
Wildtype clone #3
Wildtype clone #4
Matr3KO clone #2

Supplementary Figure 1 Generate *Matr3* KO C2C12 using CRISPR/Cas9. (a) A schematic depiction of using CRISPR/Cas9 to delete the entire Matr3 gene body to generate Matr3 KO clones (refer to method section). bi-allelic Matr3 deletion clones are screened by PCR. (b) Matr3 KO clones were confirmed by western blots. 2 independent Matr3-KO clones were used. (c) Matr3 KO clones were confirmed by immunostaining. 3 independent experiments were repeated with similar results. C2C12 cells (Day0) were immunostained with DAPI (blue) and Matr3 (red). scale bar 20μm. (d) Clonal variation in growth and differentiation among C2C12 clones. 4 representative wildtype clones and 2 Matr3-KO clones were differentiated and monitored for 4 days. Source data are provided as a Source Data file. Schematic in Supplementary Figure 1a was created with BioRender.com

Supplementary Figure 2

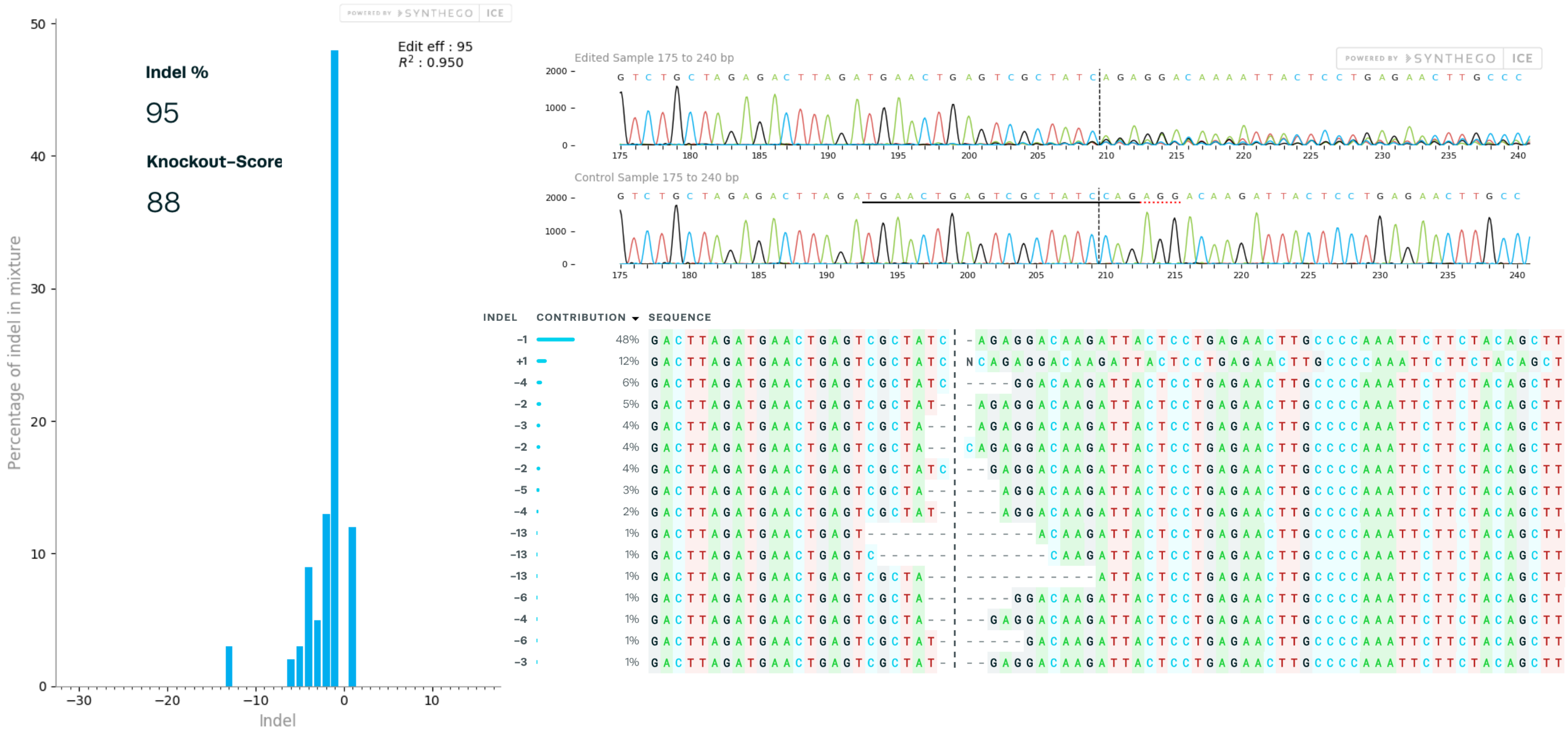

Supplementary Figure 2. Matr3 KO bulk was generated using a high-efficient guide.

Synthego ICE Sequencing report showing Matr3 KO bulk using single guide delivered as RNP had a high indel and knockout score.

a

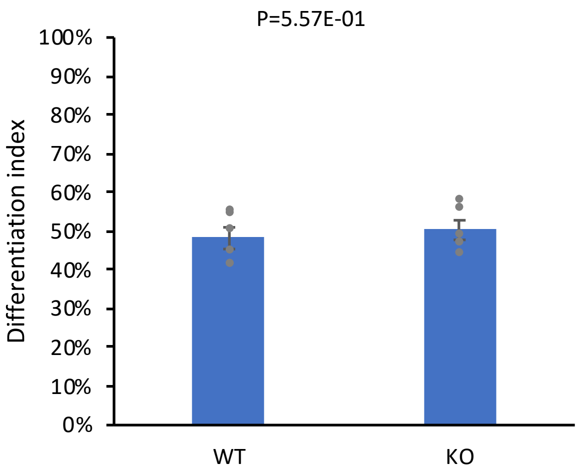
P=5.57E-01
100%
90%
80%
70%
60%
50%
40%
30%
20%
10%
0%
Differentiation index
WT
KO

b

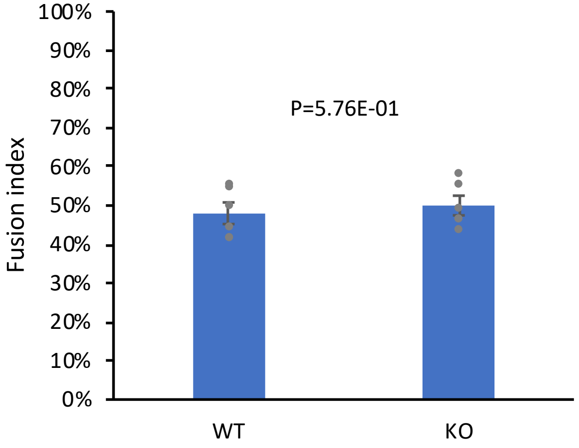
100%
90%
80%
70%
60%
50%
40%
30%
20%
10%
0%
P=5.76E-01
Fusion index
WT
KO

Supplementary Figure 3. Matr3KO accelerated myoblast differentiation and myotube fusion. (a) Quantification of the differentiation index for Figure 1c, calculated as the % nuclei in myosin heavy chain (MHC) of total nuclei (n=6 independent experiments). Unpaired two-tailed Student's t-test was used. (b) Fusion index for Figure 1c, calculated as % nuclei in MHC+ myotubes (n>3 nuclei) of total nuclei (n=6 independent experiments). Unpaired two-tailed Student's t-test. Data are presented in Figure1 as mean values +/- SEM. Source data are provided as a Source Data file.

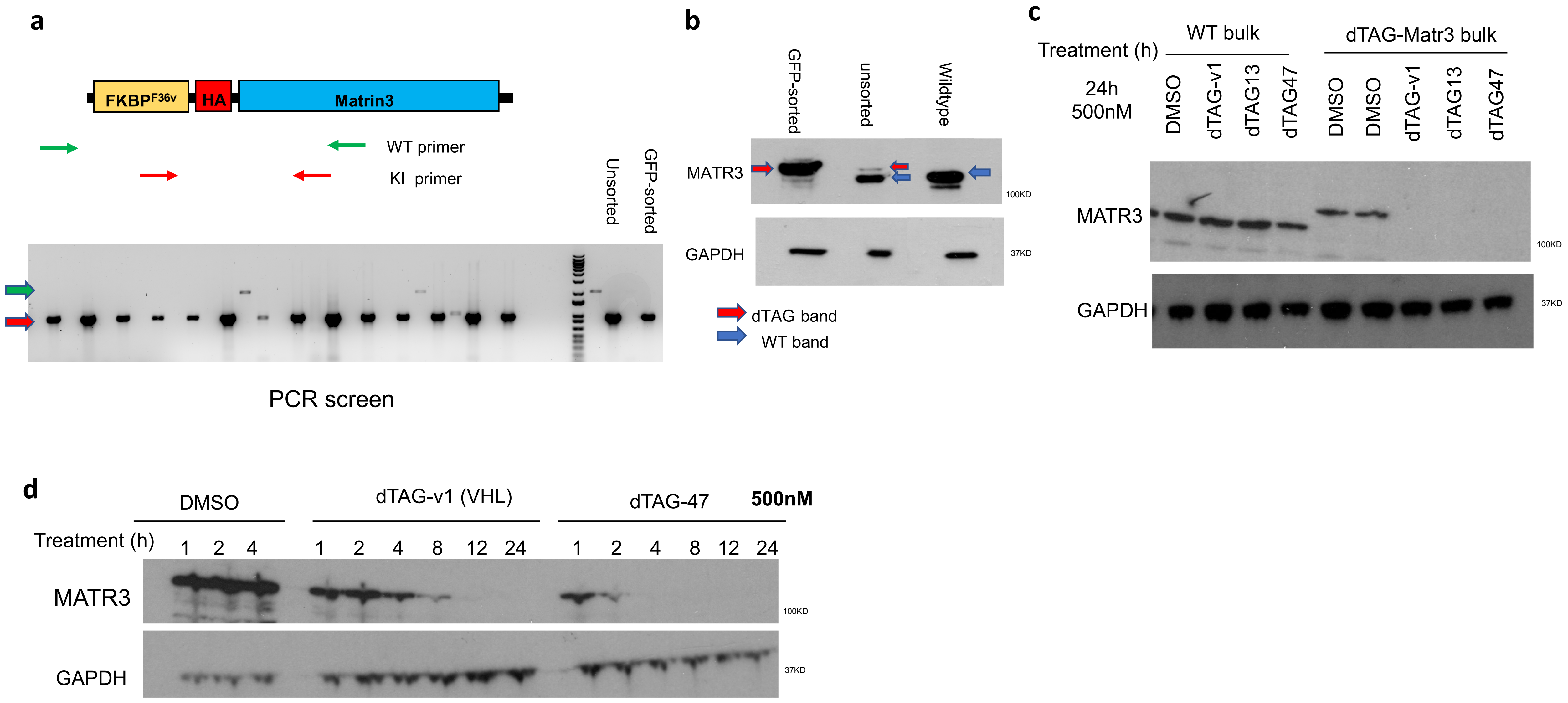
a
FKBPF36v
HA
Matrin3
WT primer
KI primer
Unsorted
GFP-sorted
PCR screen
b
GFP-sorted
unsorted
Wildtype
MATR3
GAPDH
100KD
37KD
dTAG band
WT band
c
Treatment (h)
WT bulk
dTAG-Matr3 bulk
24h
500nM
DMSO
dTAG-v1
dTAG13
dTAG47
DMSO
DMSO
dTAG-v1
dTAG13
dTAG47
MATR3
GAPDH
100KD
37KD
d
DMSO
dTAG-v1 (VHL)
dTAG-47
500nM
Treatment (h)
1 2 4
1 2 4 8 12 24
1 2 4 8 12 24
MATR3
GAPDH
100KD
37KD

Supplementary Figure 4. N-terminal dTAG-Matr3 bulk was generated and confirmed. (a) Biallelic dTAG-Matr3 knock-in clones were screened by PCR. >85% of the clones from the screening were bi-allelic dTAG-Matr3 knock-in. (b) High-efficient knock-in bulk FKBP F36v-Matr3 were sorted by GFP and confirmed by western blots. Cells enriched in the top 0.2% highest GFP signal were sorted (GFP-sorted). FKBP F36v-Matr3 (refer as dTAG-Matr3) bands were enriched in the GFP-sorted bulk (left lane), compared with unsorted bulk (middle lane), indicating the high-efficient knock-in efficiency. (c) dTAG-Matr3 protein was degraded by all 3 PROTACs (dTAG-v-1, dTAG13, dTAG47 500nM) completely after 24hrs. treatment. (d) dTAG-Matr3 in C2C12 were degraded more efficiently by dTAG47 (500nM, 4h) than dTAG-v1 (500nM, 12h). 3 independent experiments were repeated with similar results. Source data are provided as a Source Data file.

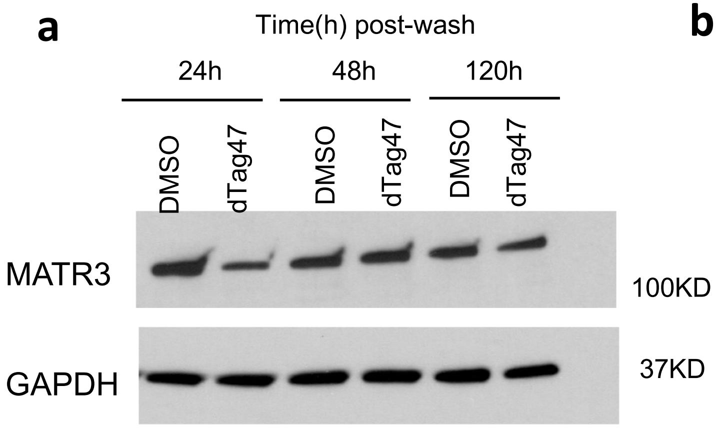
a
Time(h) post-wash
24h
48h
120h
DMSO
dTag47
DMSO
dTag47
DMSO
dTag47
MATR3
100KD
GAPDH
37KD

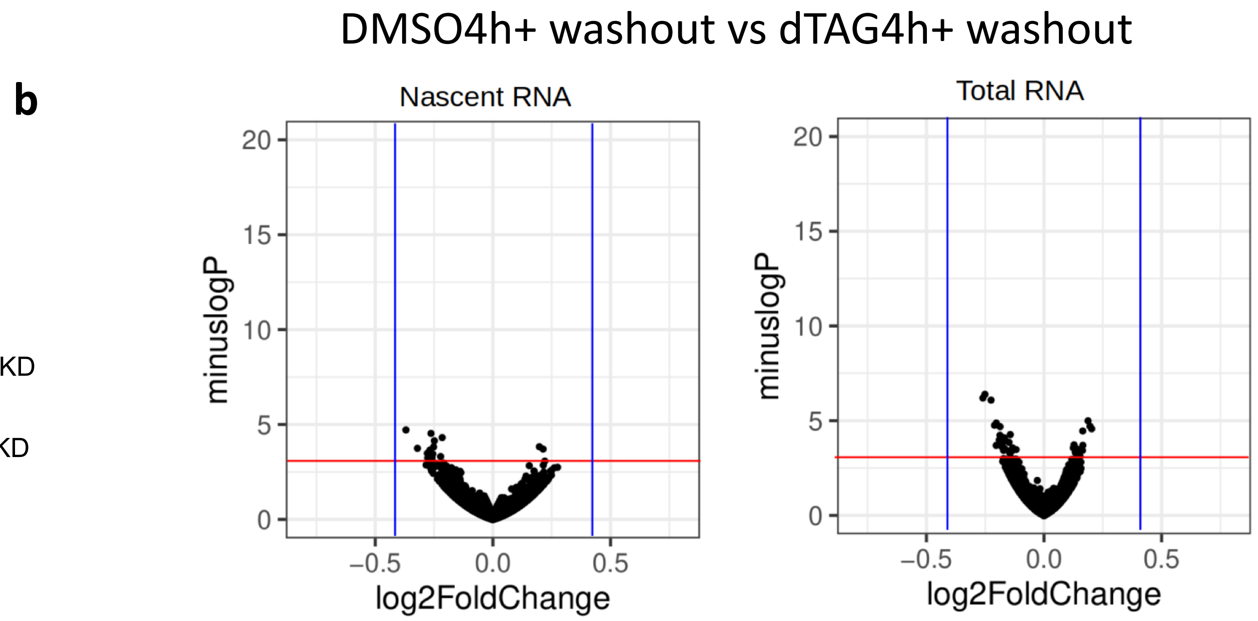
b
DMSO4h+ washout vs dTAG4h+ washout
Nascent RNA
Total RNA
minuslogP
log2FoldChange
20
15
10
5
0
−0.5
0.0
0.5

Supplementary Figure 5. Rescue of gene expression in Cells, Related to Figure 2d. (a) Western blots validation of MATR3 recovery after 4hrs. treatment with dTAG compound followed by 5 days washout of the drug. 2 independent experiments were repeated with similar results. (b) Nascent RNA and total RNA were quantified by SLAM-seq in the washout (volcano plots, p-values from the Wald test ($p<0.05$) for red line that denotes significant DE genes, and $\log_2FC>0.4$ for the vertical blue lines). There are no longer significant differences between dTAG and DMSO in the affected genes (Figure 2d). Source data are provided as a Source Data file.

Supplementary Figure 6

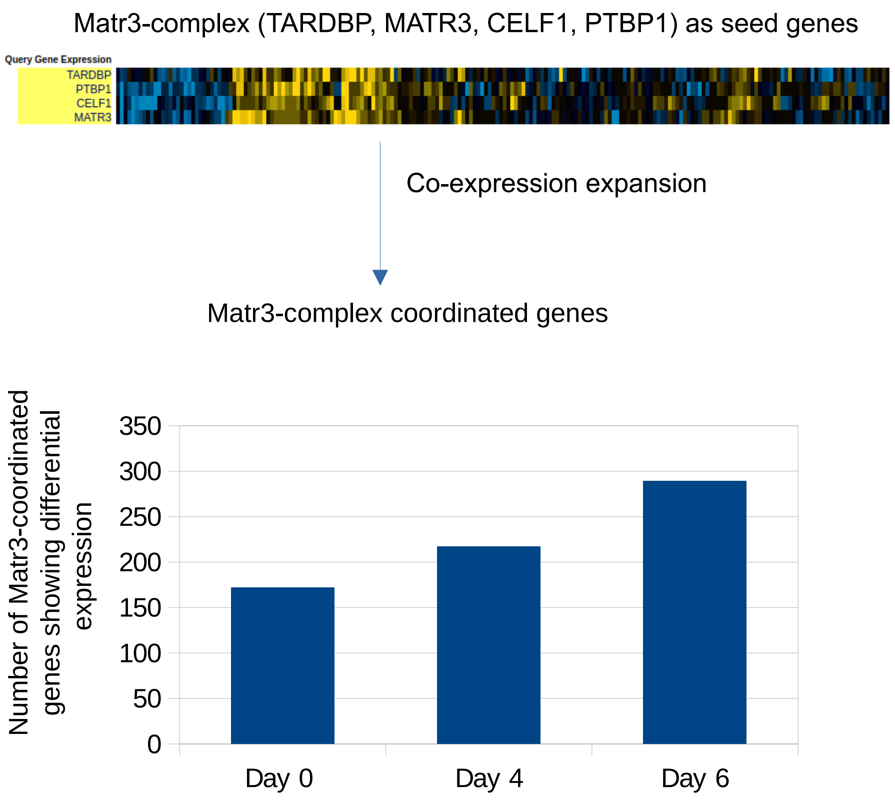

Supplementary Figure 6. Matr3 co-expressed genes are perturbed in expression at later time points. First, we used SEEK to identify Matr3-coordinated (or co-expressed genes). We entered MATR3 along with 4 other genes found in co-complex with MATR3 (TARDBP, MATR3, CELF1, PTBP1) forming a coherent query gene-set, in order to steer SEEK to use the right datasets (where MATR3 is active) for co-expression analysis. SEEK returned the co-expressed genes to the query using a large body of gene expression data sets where MATR3 and complex expression was high. The top 500 co-expressed genes were isolated and correlated with our C2C12 Day 0, Day 4, Day 6 differentiated KO vs. WT gene-sets, in order to assess what proportion of the co-expressed genes changed expression during development. As shown, Matr3 co-expressed genes were increasingly perturbed in expression over development time.

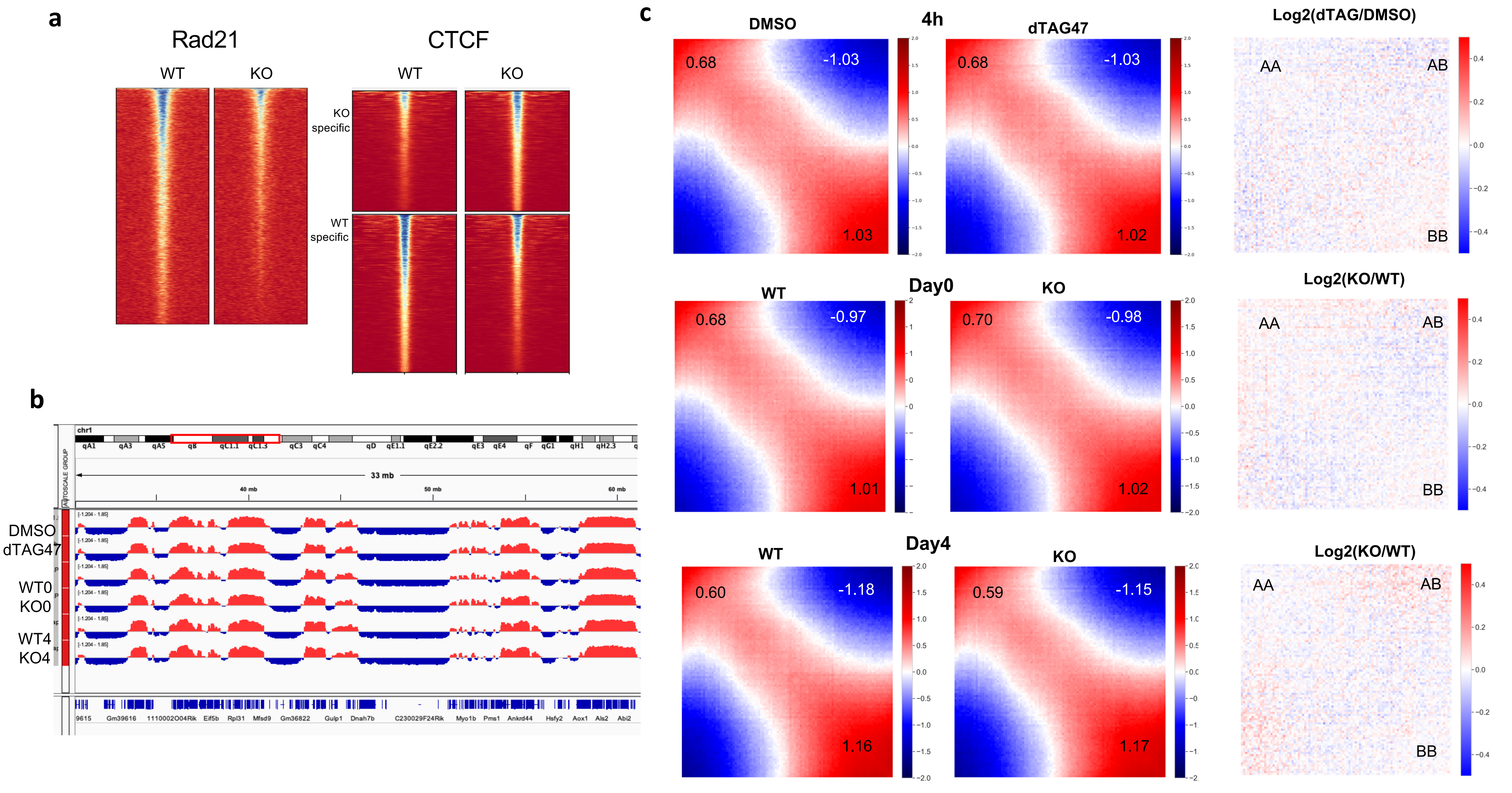
a
Rad21
WT
KO
CTCF
WT
KO
KO specific
WT specific
b
chr1
33 mb
40 mb
50 mb
60 mb
DMSO
dTAG47
WT0
KO0
WT4
KO4
c
4h
DMSO
dTAG47
0.68
-1.03
1.03
0.68
-1.03
1.02
Log2(dTAG/DMSO)
AA
AB
BB
Day0
WT
KO
0.68
-0.97
1.01
0.70
-0.98
1.02
Log2(KO/WT)
AA
AB
BB
Day4
WT
KO
0.60
-1.18
1.16
0.59
-1.15
1.17
Log2(KO/WT)
AA
AB
BB

Supplementary Figure 7. Matr3 has minimal impact on compartments switching and interaction. (a) Heatmap showing substantial reduction of Rad21 binding in Matr3 KO. CTCF occupancy was marked by both gains and losses in Matr3 KO. (b) Snapshot of compartmentalization landscapes of indicated samples at Chromosome 1. PC1 values of Hi-C data were shown, with red color representing active compartment A and blue representing inactive compartment. (c) Saddle plots showing the average interaction within and between compartments A and B. Numbers in the heatmaps indicated the average compartment strength (Log2 Obs/Exp) for intra- (A-A in top-left, B-B in bottom-right) and inter-compartment interactions (AB, top-right or bottom-left). The differential heatmap for each comparison was shown on the right panel with red/blue color representing increase/decrease in interaction strength.

Supplementary Figure 8

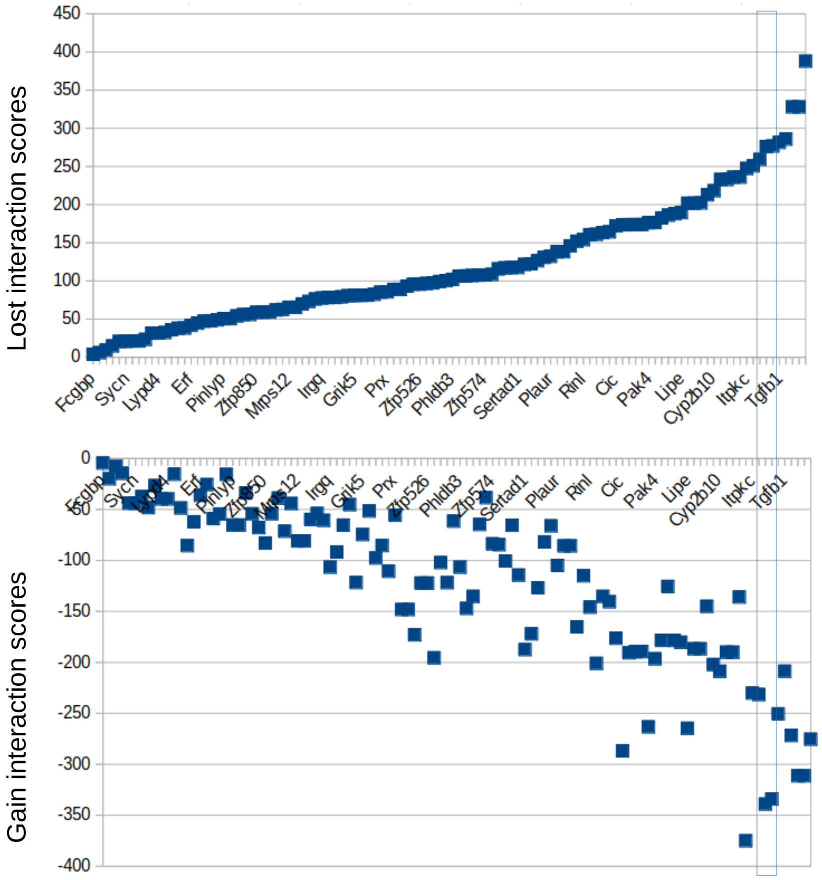

Supplementary Figure 8. Re-arranged loops were observed in the WT and KO Matr3 Hi-C experiments. Gene loci on chromosome 7 were arranged based on differential loop interaction scores as observed in the WT vs. KO Matr3 Hi-C experiments. Top: lost loop interaction scores. Bottom: gained interaction scores at each gene locus. Loop interaction scores were summarized per gene TSS location. Boxed: Tgfb1 locus was again ranked among top of the most rearranged gene loci.

Supplementary Figure 9

a

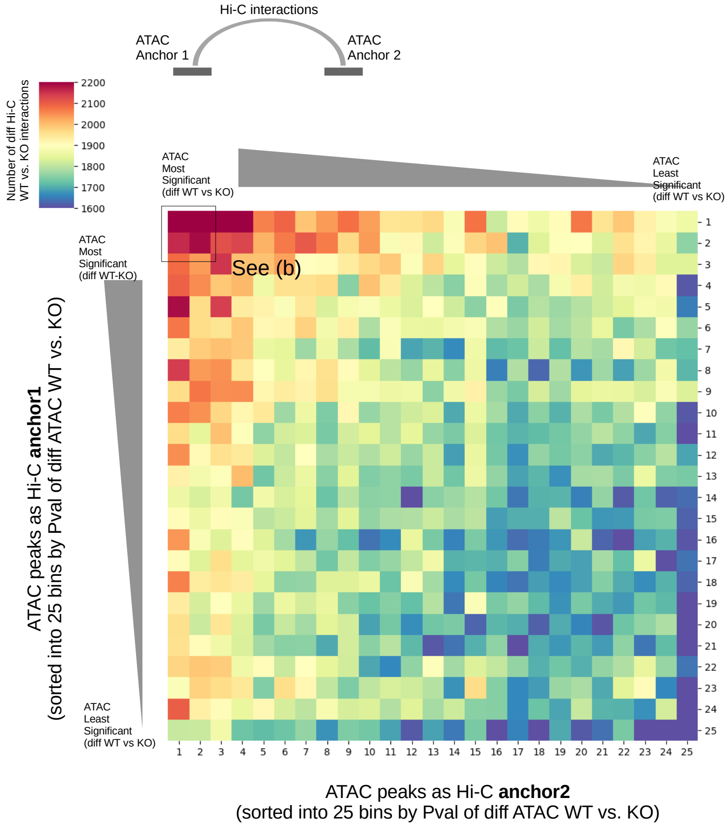

b

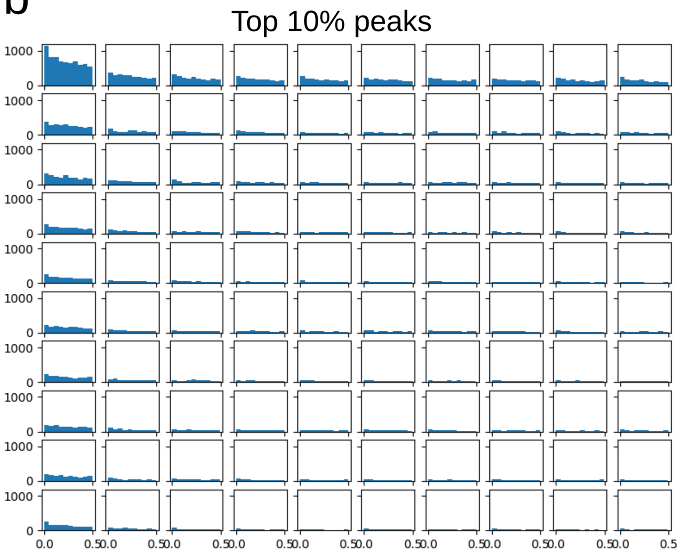

Supplementary Figure 9. Differential ATAC-seq signals are correlated with differential Hi-C interactions. (a) We interrogated the strength of Hi-C interactions for pairs of anchors overlapping with ATAC-seq peaks (termed ATAC anchor 1 and anchor 2) (see cartoon). For a pair of anchors (x, y), where x is anchor 1 and y is anchor 2, both of which overlap with ATAC-seq, we counted the number of differential Hi-C interactions (WT vs KO) happening at (x, y). ATAC anchors are sorted into 25 bins based on WT/KO ATAC-differential P-values of individual anchors (see axis titles). Thus, as result of this sorting, the top left corner of the heatmap (boxed) quantifies the number of differential Hi-C interactions between the most significant differential ATAC-seq peaks serving as anchors. (b) The top 10% fraction, divided into 10 X 1% bins. Each histogram quantified the number of differential Hi-C interactions observed in that percentile bin.

a

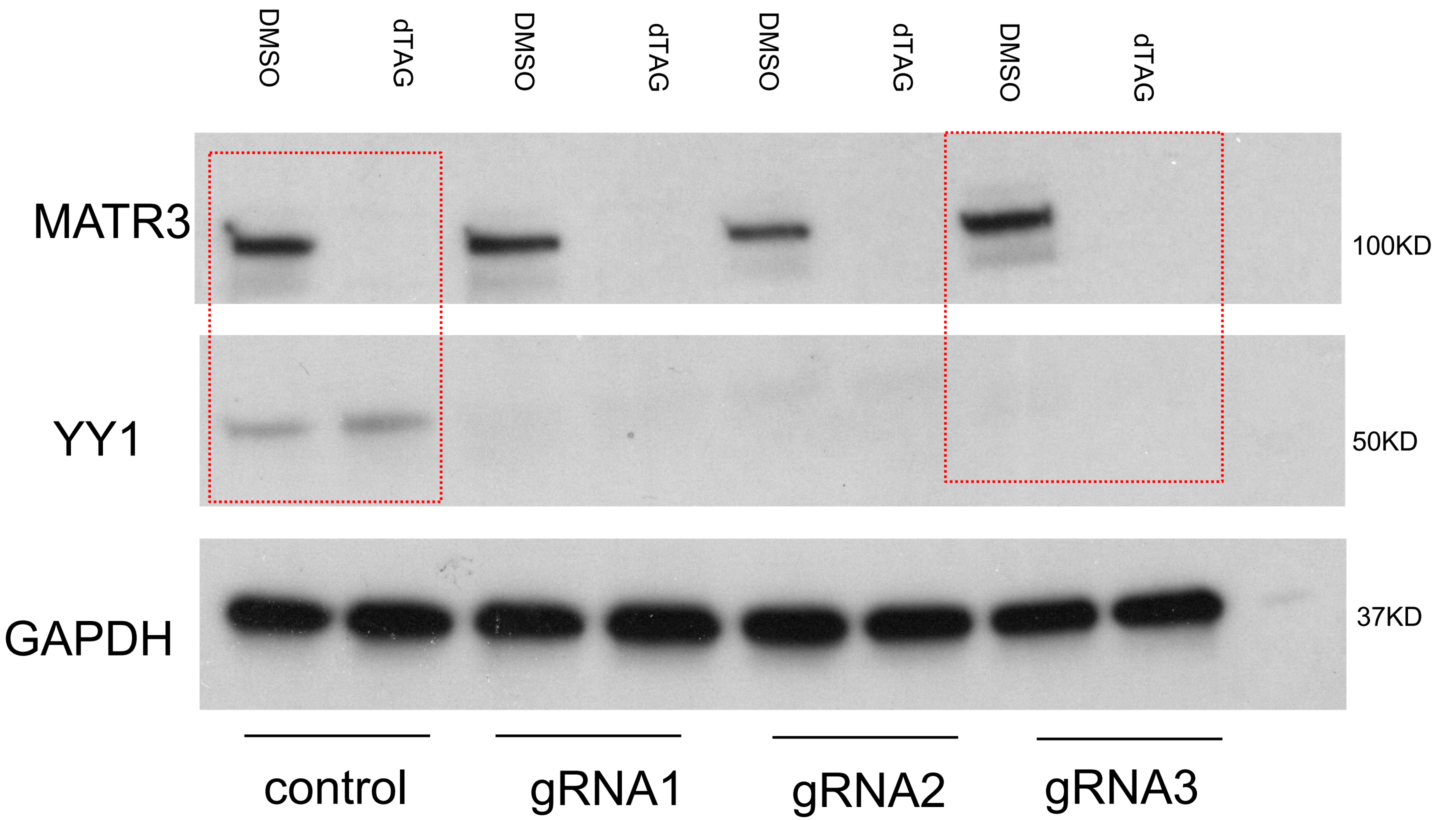
DMSO
dTAG
DMSO
dTAG
DMSO
dTAG
DMSO
dTAG
MATR3
YY1
GAPDH
100KD
50KD
37KD
control
gRNA1
gRNA2
gRNA3

b

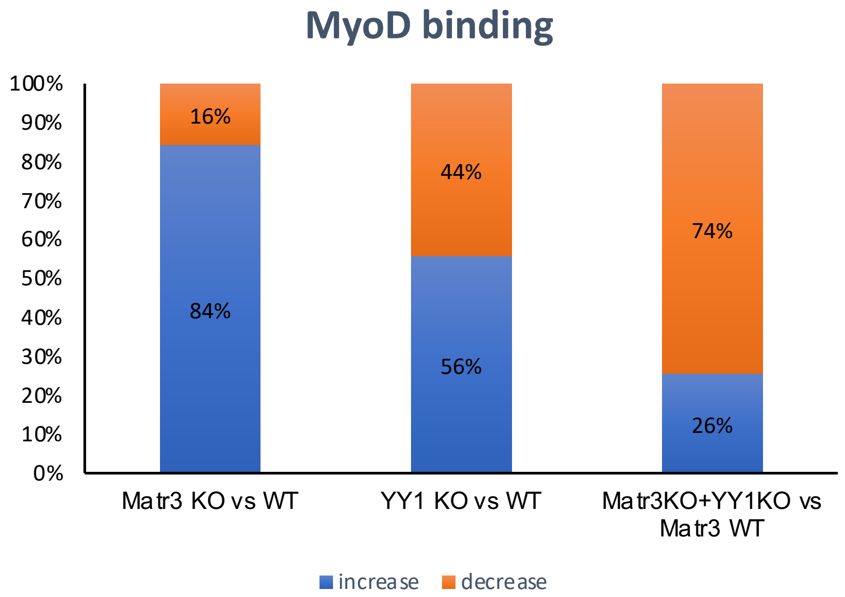
MyoD binding
100%
90%
80%
70%
60%
50%
40%
30%
20%
10%
0%
16%
84%
44%
56%
74%
26%
Matr3 KO vs WT
YY1 KO vs WT
Matr3KO+YY1KO vs Matr3 WT
increase
decrease

Supplementary Figure 10. Recruitment of MyoD upon Matr3 depletion is dependent on YY1 binding. (a) Western blots validation of MATR3 and YY1 after 4hrs. treatment with DMSO/dTAG compound in YY1 knockout (KO) +dTAG-Matr3 cells. 2 independent experiments were repeated with similar results. dTAG-Matr3 line with YY1 KO was generated by Cas9/RNP transfection (see methods). 3 different sets of gRNAs were used to electroporated into dTAG-Matr3 cells and the most efficient guide, gRNA3 was selected for all the later analysis in (b). (b) Bar chart: percentage of MyoD binding peak increased and decreased in the following condition: left plot, Matr3KO (4hrs.dTAG47 treatment) vs WT (4hrs. DMSO treatment); middle plot, YY1KO (YY1KO + 4hrs. DMSO treatment) vs WT (4hrs. DMSO treatment); right plot, Matr3KO+YY1KO (YY1KO + 4hrs. dTAG47 treatment) vs WT (4hrs. DMSO treatment). Source data are provided as a Source Data file.

a

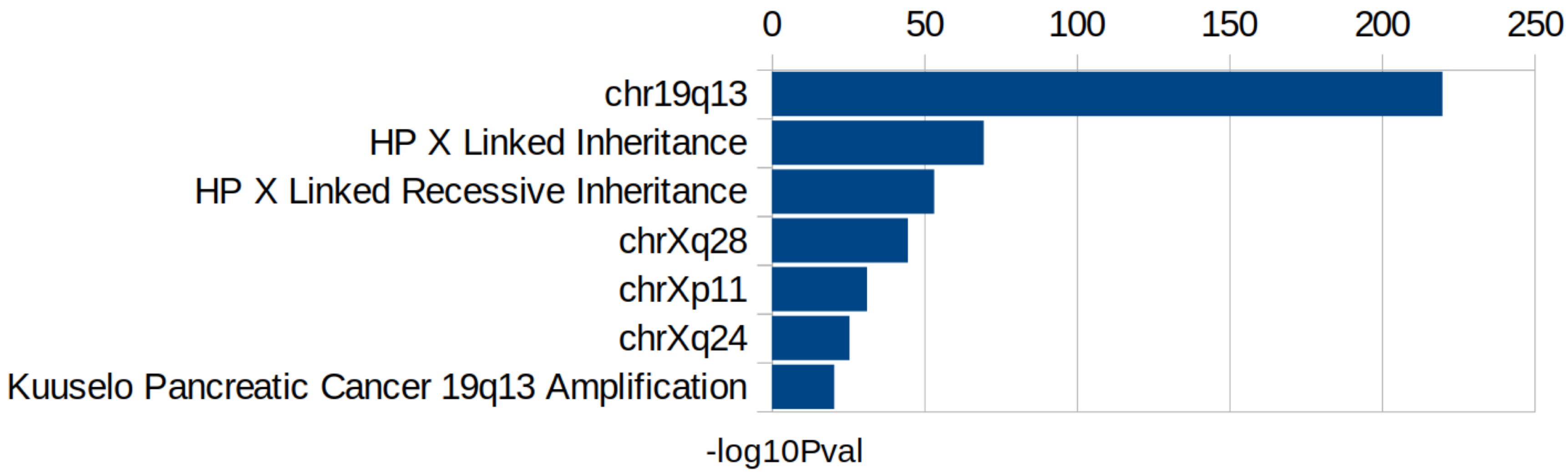
0
50
100
150
200
250
chr19q13
HP X Linked Inheritance
HP X Linked Recessive Inheritance
chrXq28
chrXp11
chrXq24
Kuuselo Pancreatic Cancer 19q13 Amplification
-log10Pval

b

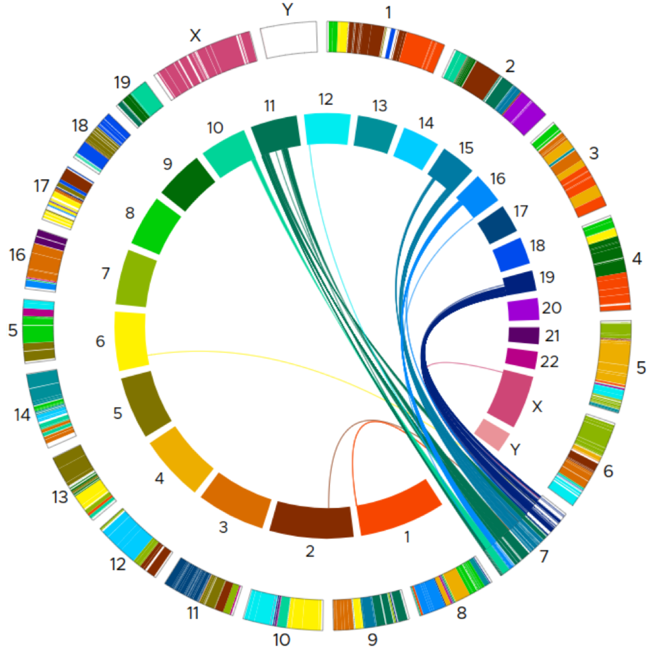
Y
X
1
2
3
4
5
6
7
8
9
10
11
12
13
14
15
16
17
18
19
20
21
22

Supplementary Figure 11. Rearranged loop domains by loss of Matr3 are concentrated at the conserved hotspots related to cancer and muscular disease. ~500 genes located in rearrangement hotspots (in chrs 14, 7, X) were collected and used for GSEA analysis on MsigDB concepts. (a) Indicated are the top ranked human chromosomal cytobands, and the disease ontology concept terms enriched for the 500 genes. (b) It should be noted that chr19q13 (the top enriched term) is a conserved segment to mouse chr7 segment. Outer circle indicates mouse chromosomes. Inner circle indicates human chromosomes. The rearranged hotspots on chr7 align only to chr19 in human.

a Loop calling at various resolution with Hiccup algorithm

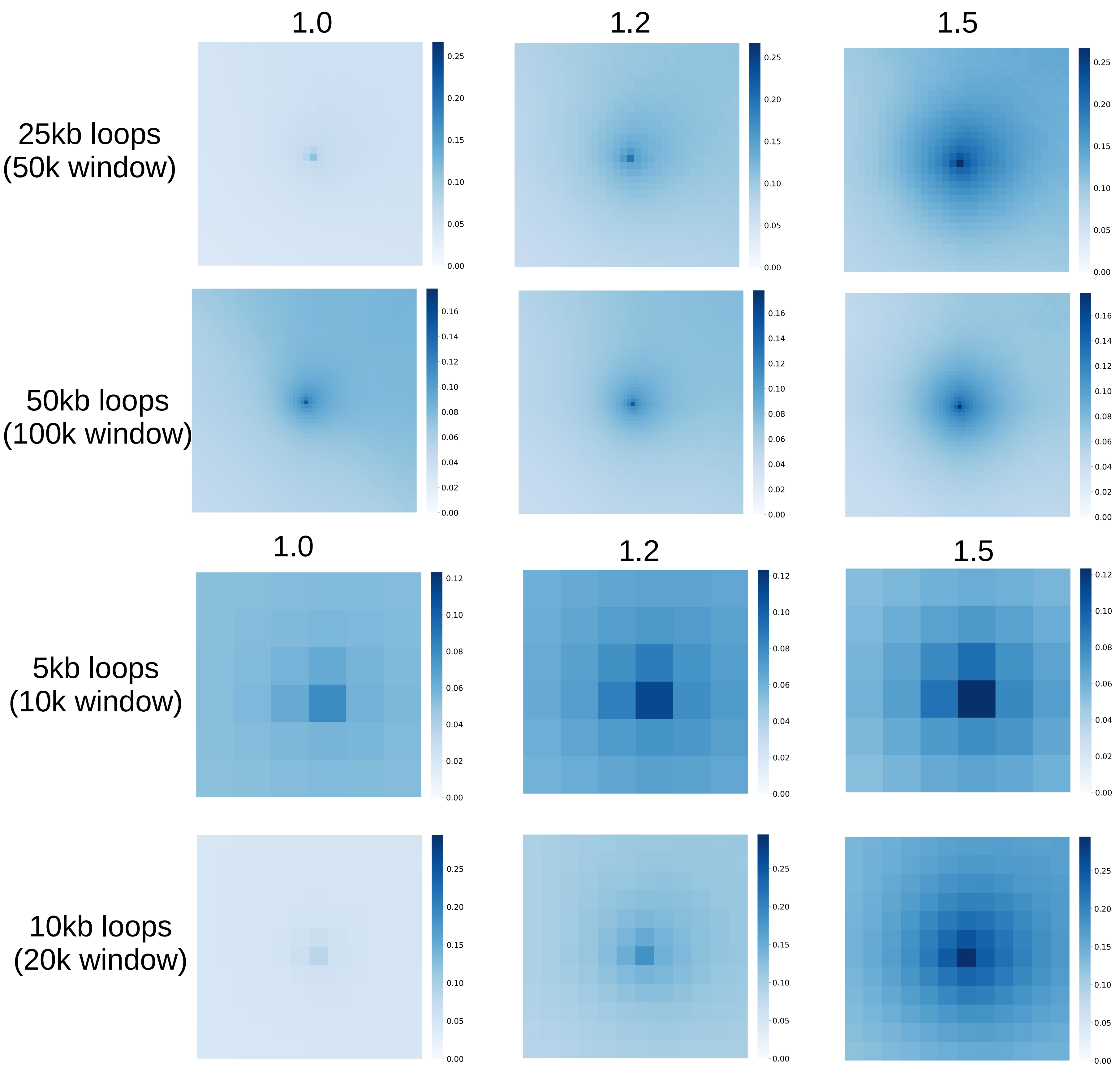

b Loop calling comparison, agreement between Hiccup and FitHiC with or without HIF imputation

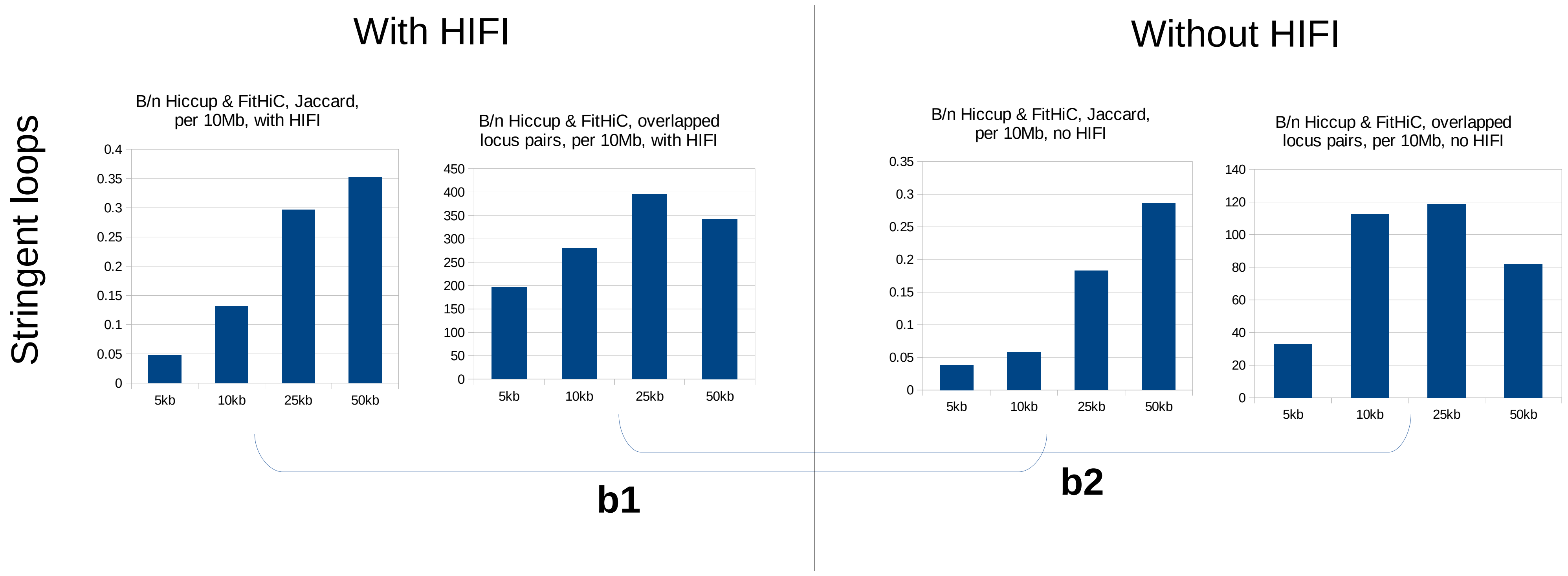

Supplementary Figure 12. Loop calling with HIFI pre-processing. (**a**) Loop calling at various resolutions and various levels of stringencies using Hiccup algorithm, with HIFI-based preprocessing. 5kb, 10kb, 25kb loops and 50kb loops were visualized within 10 kb-, 20 kb-, 50 kb- and 100 kb-sized windows respectively. Three stringencies of loops (1.0, 1.2, and 1.5 as determined by Hiccup score) were selected for visualization. (**b**) Loop calling comparison, showing agreement between Hiccup and FitHiC with and without HIFI imputation. (**b1**) With HIFI imputation, the overlap (by Jaccard coefficient) between Hiccup and FitHiC detected loops increased from 6% to 13% (for 10kb loops), 17% to 30% (for 25kb loops), and 27% to 35% (for 50kb loops). (**b2**) With HIFI imputation, the number of agreed, detected loops per 10Mb also increased from 30 to 200 (for 5kb), 110 to 270 (for 10kb), 120 to 400 (for 25kb), and 80 to 350 (for 50kb), representing on average 3-5-fold increase.

Supplement Table 1. dTag24h vs DMSO 24h DE genes from RNA-seq

id	baseMean	log2FoldChange	stat	pvalue	padj
GM38403	310.8735	-2.030715318	-10.96386666	5.70E-28	8.06E-24
H2-DMA	301.06794	-1.4046967	-8.236786325	1.77E-16	1.25E-12
C920009B1	529.08197	1.144254713	8.086874979	6.12E-16	2.88E-12
GSTA4	3587.9	0.940813261	8.045239254	8.61E-16	3.04E-12
ASB3	444.36148	-1.230430911	-8.011714214	1.13E-15	3.20E-12
DCAF8	4332.2473	-0.787006101	-6.523332609	6.88E-11	1.62E-07
NQO1	1474.4842	0.766247067	6.338023097	2.33E-10	4.70E-07
KCNQ4	1290.6484	-0.781050356	-6.166291361	6.99E-10	1.24E-06
THUMPD2	164.3073	-1.307217397	-5.8300434	5.54E-09	7.83E-06
GSTA2	104.05708	1.649762709	5.834102323	5.41E-09	7.83E-06
RTP4	68.618505	-1.90645499	-5.784889683	7.26E-09	9.32E-06
OPN3	356.28821	-0.982742008	-5.720072061	1.06E-08	1.25E-05
LRRCC1	1641.1233	0.737531849	5.646618232	1.64E-08	1.78E-05
WDR78	79.222048	-1.881254691	-5.586423598	2.32E-08	2.34E-05
SH2D4B	20.258633	-3.498595068	-5.490523263	4.01E-08	3.78E-05
EPS15L1	2149.4304	0.577120166	5.160472357	2.46E-07	0.000197
R3HDML	411.73279	-0.900187636	-5.156892782	2.51E-07	0.000197
ACTC1	26127.818	-0.632561896	-5.170447937	2.34E-07	0.000197
MYL9	737.60101	-0.698588535	-5.130815485	2.88E-07	0.000215
TNC	1360.6222	-0.819378869	-4.963052718	6.94E-07	0.00049
ASCC3	1660.3193	-0.734074743	-4.926683178	8.36E-07	0.000563
SYNPR	308.44197	-1.037227045	-4.881740941	1.05E-06	0.000676
ARSK	341.16446	-0.751936633	-4.867281484	1.13E-06	0.000695
NR2C1	900.67179	0.628009058	4.788981266	1.68E-06	0.000934
NAA25	2302.4653	-0.64509117	-4.799843108	1.59E-06	0.000934
ALDH3A1	1777.7421	0.630099684	4.783965992	1.72E-06	0.000934
GRIA1	54.382168	-1.542377208	-4.749686884	2.04E-06	0.001066
PPP1R3F	107.58904	-1.204859966	-4.734918308	2.19E-06	0.001106
GM20139	25.310315	-2.355622641	-4.715422718	2.41E-06	0.001176
ITGB6	2092.5078	-0.608231572	-4.643263968	3.43E-06	0.001616
GPRASP1	511.10691	-0.735664509	-4.616622591	3.90E-06	0.001778
ADGRG1	558.97378	-0.775803809	-4.607801137	4.07E-06	0.001797
CLCA3B	813.91599	0.700315626	4.529103228	5.92E-06	0.002537
MTTP	1253.6282	-0.502715039	-4.393549725	1.12E-05	0.004635
HECTD2OS	249.37135	-0.781003995	-4.309260002	1.64E-05	0.006614
ACTA2	64455.187	-0.468330338	-4.24616746	2.17E-05	0.008533
CSF2RB	978.08484	0.549450519	4.240132332	2.23E-05	0.008533
ALDH3B3	34.973103	-1.713848686	-4.209421573	2.56E-05	0.009522
CLCA3A2	115.20797	0.976520722	4.171955166	3.02E-05	0.010944
STC1	167.57097	-0.993225524	-4.141274869	3.45E-05	0.012203
PTN	497.48209	-0.654165741	-4.131689766	3.60E-05	0.012413

USP11	824.16763	0.522646708	4.086682704	4.38E-05	0.014725
PPP1R12B	412.70862	-0.637487471	-4.056707572	4.98E-05	0.016358
MAL	1049.2669	0.481986755	4.048590504	5.15E-05	0.016551
SLC5A5	88.992516	-1.013422948	-4.034858022	5.46E-05	0.017159
CLIC5	674.34571	0.601104842	3.999480578	6.35E-05	0.019504
SERINC2	1671.4816	-0.51062103	-3.987825481	6.67E-05	0.020051
ADH7	1006.436	0.530185036	3.880652667	0.000104177	0.029825
GADD45G	1036.1198	-0.58399419	-3.876857131	0.000105814	0.029825
ANPEP	2270.083	0.542061022	3.88148008	0.000103823	0.029825
GM14827	7.3746824	-5.287688309	-3.872725884	0.000107625	0.029825
DLG2	81.329247	-1.014760155	-3.846415297	0.000119859	0.030799
RDH5	355.53672	-0.621155032	-3.854357028	0.000116034	0.030799
SERP2	142.10077	-0.986957816	-3.848611481	0.000118789	0.030799
CKM	8112.4344	-0.406869298	-3.858903525	0.000113897	0.030799
VWA8	1661.9888	-0.531326329	-3.83164237	0.000127291	0.032125
CPQ	2356.8543	-0.436349083	-3.81209926	0.000137792	0.033576
CACNA1B	90.868072	-0.955183719	-3.813303265	0.000137122	0.033576
TMEM182	3146.3066	-0.423537022	-3.805514779	0.00014151	0.033898
TTC19	1707.4758	-0.438993696	-3.801201304	0.000143996	0.033918
MDGA1	12.063818	-2.878783812	-3.755906551	0.000172715	0.039371
6030443J06	95.323438	0.986683234	3.757765083	0.000171438	0.039371
ENPP3	365.89431	0.621936072	3.726176039	0.000194407	0.043612
FAM169B	27.05209	-1.697210075	-3.71833362	0.000200541	0.044285
F3	771.60229	-0.470604691	-3.713070112	0.00020476	0.044521
EPDR1	1686.0009	-0.457741649	-3.704135128	0.000212113	0.045421
ZIM1	4179.0745	0.46101934	3.682620239	0.000230849	0.047979
UTP14B	1259.5564	0.437710156	3.685955993	0.000227846	0.047979

Supplementary Table 1. dTag24h vs DMSO 24h DE genes from RNA-seq. dTag-Matr3 cells were collected after 24hrs. treatment of DMSO (500nM) or dTAG47 (500nM). RNA was extracted and used for RNA-seq analysis (n=3 independent biological experiments). p-values from the Wald test less than 0.05 ($p<0.05$) and log2FC>0.4 denote significant DE genes.

Supplementary Table of primers and antibodies used in this study

Primers list			
**Reagent type**	**Name**	**Sequence / Cat #**	**Purpose**
gRNA	Matr3-KO-F1L	CACCGAGGCACGTGACGTACGCGGC	Matr3 knockout clones: single guide RNAs (sgRNAs) for targeting Matr3 coding region
gRNA	Matr3-KO-F1RC	AAACGCCGCGTACGTCACGTGCCTC	Matr3 knockout clones: single guide RNAs (sgRNAs) for targeting Matr3 coding region
gRNA	Matr3-KO-R2L	CACCGATCGGGTTTTATCGAGGTGA	Matr3 knockout clones: single guide RNAs (sgRNAs) for targeting Matr3 coding region
gRNA	Matr3-KO-R2RC	AAACTCACCTCGATAAAACCCGATC	Matr3 knockout clones: single guide RNAs (sgRNAs) for targeting Matr3 coding region
primers	Matr3-Int-L	TGAGGGCAGGATCAAGCTTT	Primers for screen Matr3 knockout clones (wildtype only)
primers	Matr3-Int-R	ATGTCAAAGCTTTCCACATGG	Primers for screen Matr3 knockout clones (wildtype only)
primers	Matr3-Ext-L	TTCCTTGCCTTCTCTGAGGC	Primers for screen Matr3 knockout clones (knock out only)
primers	Matr3-Ext-R	CAGCACTGGGTTGGACATCT	Primers for screen Matr3 knockout clones (knock out only)
modified-sgRNA	sgRNA-Matr3RNP	UGAACUGAGUCGCUAUCCAG	modified-sgRNA for generating Matr3 knockout bulk
primers	Matr3_RNP_F1	ATGAACCAGGGTACTGCACG	for PCR to check RNP knockout efficiency
primers	Matr3_RNP_R1	GCATCTGTTGCTTTAACTCACCTT	for PCR to check RNP knockout efficiency
primers	Matr3_RNP_F2	TCGGTAGGGATTCACAGGGT	for PCR to check RNP knockout efficiency
primers	Matr3_RNP_R2	AGCATCTGTTGCTTTAACTCACCT	for PCR to check RNP knockout efficiency
sequence primer	Seq primer RNP	ACTGCACGCCTTGCTAGTTT	sequencing primer for check RNP knockout efficiency

modified-sgRNA	sgRNA-N-dTag-Matr3	UCUCUCGGUAGGGAUUCACA	modified-sgRNA for generating knockin N-terminal dTag-Matr3
primers	TL_Ntag_WT_F2	GCTAACTTGGGATGTTTCCCTT	Primers for screen Matr3 knockout clones (wildtype only)
primers	TL_Ntag_WT_R2	GTGGCAAGAAGCATTGTAGGT	Primers for screen Matr3 knockout clones (wildtype only)
primers	TL_Ntag_KI_F1	GCTACCCCGACCACATGAAG	Primers for screen dTag Matr3 knockin clones (knock in only)
primers	TL_Ntag_KI_R1	CCGGGACATCATACGGATAGC	Primers for screen dTag Matr3 knockin clones (knock in only)
modified-sgRNA	sgRNA-YY1RNP-1	GCCCACCACCGUGGUCUCGA	modified-sgRNA for generating YY1 knockout bulk
modified-sgRNA	sgRNA-YY1RNP-2	ACCCUCUACAUCGCCACGGA	modified-sgRNA for generating YY1 knockout bulk
modified-sgRNA	sgRNA-YY1RNP-3-1	UGCAGCUCCACGAUCUCGGC	modified-sgRNA multiguide for generating YY1 knockout bulk
modified-sgRNA	sgRNA-YY1RNP-3-2	GCCCACCACCGUGGUCUCGA	modified-sgRNA multiguide for generating YY1 knockout bulk
modified-sgRNA	sgRNA-YY1RNP-3-3	GGUGGUGGCCGGCGUGCCCG	modified-sgRNA multiguide for generating YY1 knockout bulk

Antibody list		
**Name**	**Cat #**	**Purpose**
Anti-Matrin3	anti-Matrin3	proteintech, 12202-2-AP
anti-Matrin3	Sigma HPA036565	1:1000 for WB
anti-Matrin3	abcam151739	1:200 for IF
anti-DMD	a gift from Kunkel Laboratory	1:500 for IF, 1:1000 for WB
anti-DCAF8	Bethyl Laboratories® Catalog # A301-556A	1:500 for IF, 1:5000 for WB
anti-myosin heavy chain	DSHB Hybridoma Product MF 20, deposited to the DSHB by Fischman, D.A	1:40 for IF, 1:400 for WB
anti-GAPDH	abcam: ab9485	1:10,000 for WB
Goat Anti-Mouse IgG (H + L)-HRP Conjugate	BIO-RAD, #170-6516	1:2000 for WB
Goat Anti-Rabbit IgG (H+ L)-HRP Conjugate	BIO-RAD, #170-6515	1:10,000 for WB
anti-HA-Tag (C29F4) Rabbit mAb	cell signaling, HA-Tag (C29F4) Rabbit mAb #3724	1:1000 for WB
Alex488 donkey anti-rabbit	Jackson ImmunoResearch	1:200 for IF
Alex647 donkey anti-mouse	Jackson ImmunoResearch	1:200 for IF
anti-CTCF	Abcam, ab70303	5 -10μg antibody per ChIP reaction
anti-CTCF	Millipore 07-729	1:100 for CUT&RUN
anti-Rad21	Abcam, ab992	5 -10μg antibody per ChIP reaction, 1:100 for CUT&RUN
MyoD	Santa cruz, Anti-MyoD Antibody (5.8A), sc-32758 X	1:100 for CUT&RUN
anti-YY1	santa cruz, sc-7341X	1:100 for CUT&RUN
Histone H3K4me3 antibody (pAb)	active motif, 39159	1:50 for CUT&RUN
Anti-acetyl-Histone H3 (Lys27) Antibody, clone RM172	millipore, MABE647	1:50 for CUT&RUN
Rabbit Anti-Mouse IgG H&L	abcam, ab46540	1:100 for secondary antibody in CUT&RUN (primary antibody from mouse)
