# Supplementary material for: Matrin3 mediates differentiation through stabilizing chromatin loop-domain interactions and YY1 mediated enhancer-promoter interactions
Source: Nat Commun. 2024 Feb 10;15:1274. doi: 10.1038/s41467-024-45386-w (PMC10858947; doi:10.1038/s41467-024-45386-w)
Supplement: Supplementary file 3 — Reporting Summary [file 41467_2024_45386_MOESM3_ESM.pdf]

nature portfolio

Corresponding author(s): Stuart H Orkin

Last updated by author(s): 12/18/2023

Reporting Summary

Nature Portfolio wishes to improve the reproducibility of the work that we publish. This form provides structure for consistency and transparency in reporting. For further information on Nature Portfolio policies, see our Editorial Policies and the Editorial Policy Checklist.

Statistics

For all statistical analyses, confirm that the following items are present in the figure legend, table legend, main text, or Methods section.

n/a	Confirmed	
☐	☒	The exact sample size (*n*) for each experimental group/condition, given as a discrete number and unit of measurement
☐	☒	A statement on whether measurements were taken from distinct samples or whether the same sample was measured repeatedly
☐	☒	The statistical test(s) used AND whether they are one- or two-sided<br>*Only common tests should be described solely by name; describe more complex techniques in the Methods section.*
☒	☐	A description of all covariates tested
☐	☒	A description of any assumptions or corrections, such as tests of normality and adjustment for multiple comparisons
☐	☒	A full description of the statistical parameters including central tendency (e.g. means) or other basic estimates (e.g. regression coefficient) AND variation (e.g. standard deviation) or associated estimates of uncertainty (e.g. confidence intervals)
☐	☒	For null hypothesis testing, the test statistic (e.g. *F*, *t*, *r*) with confidence intervals, effect sizes, degrees of freedom and *P* value noted<br>*Give P values as exact values whenever suitable.*
☒	☐	For Bayesian analysis, information on the choice of priors and Markov chain Monte Carlo settings
☒	☐	For hierarchical and complex designs, identification of the appropriate level for tests and full reporting of outcomes
☒	☐	Estimates of effect sizes (e.g. Cohen's *d*, Pearson's *r*), indicating how they were calculated

Our web collection on statistics for biologists contains articles on many of the points above.

Software and code

Policy information about availability of computer code

Data collection	CV7000 software to collect Yokogawa CV7000 microscope imaging
Data analysis	bedtools (2.27.1) https://academic.oup.com/bioinformatics/article/26/6/841/244688<br>samtools (1.15.1) https://academic.oup.com/bioinformatics/article/25/16/2078/204688<br>macs2 (2.1.1) https://genomebiology.biomedcentral.com/articles/10.1186/gb-2008-9-9-r137<br>DESeq2 (1.14.1) https://genomebiology.biomedcentral.com/articles/10.1186/s13059-014-0550-8<br>CUT&RUNTools (1.0) https://genomebiology.biomedcentral.com/articles/10.1186/s13059-019-1802-4<br>bowtie2 (2.2.9) https://www.ncbi.nlm.nih.gov/pmc/articles/PMC3322381/<br>HIFI (1.0) https://genomebiology.biomedcentral.com/articles/10.1186/s13059-019-1913-y<br>deeptools (3.0.2) https://www.ncbi.nlm.nih.gov/pmc/articles/PMC4086134/<br>Slamdunk (1.0) https://bmcbioinformatics.biomedcentral.com/articles/10.1186/s12859-019-2849-7<br>bamtools (2.5.1) https://academic.oup.com/bioinformatics/article/27/12/1691/255399<br>Trimmomatic (0.36) https://www.ncbi.nlm.nih.gov/pmc/articles/PMC4103590/<br>HiC-Pro (2.10.0) https://genomebiology.biomedcentral.com/articles/10.1186/s13059-015-0831-x<br>bedops (2.4.30) https://academic.oup.com/bioinformatics/article/28/14/1919/218826<br>Hiccup (1.6) (https://www.cell.com/fulltext/S2405-4712(16)30219-8<br>ImageJ (version 1.45f)<br>Python (version 3.85)<br>pandas (version 1.1.3)<br>Seaborn (version 0.11.0)

matplotlib (version 3.3.2).
scipy (version 1.7.3)

For manuscripts utilizing custom algorithms or software that are central to the research but not yet described in published literature, software must be made available to editors and reviewers. We strongly encourage code deposition in a community repository (e.g. GitHub). See the Nature Portfolio guidelines for submitting code & software for further information.

Data

Policy information about availability of data

All manuscripts must include a data availability statement. This statement should provide the following information, where applicable:

- Accession codes, unique identifiers, or web links for publicly available datasets
- A description of any restrictions on data availability
- For clinical datasets or third party data, please ensure that the statement adheres to our policy

The data that support this study are available from the corresponding author upon
reasonable request. Hi-C, CUT&RUN, ChIP-seq, ATAC-seq, RNA-seq and SLAM-seq data sets generated this study have been deposited in the GEO database, under accession code GSE247105. Source data are provided with this paper.

Research involving human participants, their data, or biological material

Policy information about studies with human participants or human data. See also policy information about sex, gender (identity/presentation), and sexual orientation and race, ethnicity and racism.

Reporting on sex and gender	N/A
Reporting on race, ethnicity, or other socially relevant groupings	N/A
Population characteristics	N/A
Recruitment	N/A
Ethics oversight	N/A

Note that full information on the approval of the study protocol must also be provided in the manuscript.

Field-specific reporting

Please select the one below that is the best fit for your research. If you are not sure, read the appropriate sections before making your selection.

☒ Life sciences ☐ Behavioural & social sciences ☐ Ecological, evolutionary & environmental sciences

For a reference copy of the document with all sections, see nature.com/documents/nr-reporting-summary-flat.pdf

Life sciences study design

All studies must disclose on these points even when the disclosure is negative.

Sample size	Sample sizes was chosen based on published data using similar approaches. https://pubmed.ncbi.nlm.nih.gov/32817427/; https://pubmed.ncbi.nlm.nih.gov/28841410/
Data exclusions	No data were excluded.
Replication	All experiments were reliably repeated at least two times, and all the attempts at replication were successful.
Randomization	Experimental groups of cells were assigned according to their genotype, time of drug treatment and development stages
Blinding	Imaging data collection was made blinded to group allocation. Blinding was not relevant to the genomic data because investigator's bias would not affect the data collection. Data analysis was not blinded because it is strictly quantitative.

Reporting for specific materials, systems and methods

We require information from authors about some types of materials, experimental systems and methods used in many studies. Here, indicate whether each material, system or method listed is relevant to your study. If you are not sure if a list item applies to your research, read the appropriate section before selecting a response.

Materials & experimental systems

n/a	Involved in the study	
☐	☒	Antibodies
☐	☒	Eukaryotic cell lines
☒	☐	Palaeontology and archaeology
☒	☐	Animals and other organisms
☒	☐	Clinical data
☒	☐	Dual use research of concern
☒	☐	Plants

Methods

n/a	Involved in the study	
☐	☒	ChIP-seq
☐	☒	Flow cytometry
☒	☐	MRI-based neuroimaging

Antibodies

Antibodies used	anti-Matrin3 proteintech, 12202-2-AP 1:40 for CUT&RUN<br>anti-Matrin3 Sigma HPA036565 1:1000 for WB<br>anti-Matrin3 abcam151739 1:200 for IF<br>anti-DMD a gift from Kunkel Laboratory 1:500 for IF, 1:1000 for WB<br>anti-DCAF8 Bethyl Laboratories® Catalog # A301-556A 1:500 for IF, 1:5000 for WB<br>anti-myosin heavy chain DSHB Hybridoma Product MF 20, deposited to the DSHB by Fischman, D.A 1:40 for IF, 1:400 for WB<br>anti-GAPDH abcam: ab9485 1:10,000 for WB<br>anti-HA-Tag (C29F4) Rabbit mAb cell signaling, HA-Tag (C29F4) Rabbit mAb #3724 1:1000 for WB<br>anti-CTCF Abcam, ab70303 5 -10µg antibody per ChIP reaction<br>anti-CTCF Millipore 07-729 1:100 for CUT&RUN<br>anti-Rad21 Abcam, ab992 5 -10µg antibody per ChIP, 1:100 for CUT&RUN<br>MyoD Santa cruz, Anti-MyoD Antibody (5.8A), sc-32758 X 1:100 for CUT&RUN<br>anti-YY1 santa cruz, sc-7341X 1:100 for CUT&RUN<br>Histone H3K4me3 antibody (pAb) active motif, 39159 1:50 for CUT&RUN<br>Anti-acetyl-Histone H3 (Lys27) Antibody, clone RM172 millipore, MABE647 1:50 for CUT&RUN
Validation	anti-Matrin3 proteintech, 12202-2-AP for CUT&RUN, PMID: 37000624.<br>anti-Matrin3 Sigma HPA036565, western blots, https://www.sigmaaldrich.com/US/en/coa/SIGMA/HPA036565/R33715<br>anti-Matrin3 abcam151739 for IF, https://www.abcam.com/products/primary-antibodies/matrin-3-antibody-epr10634b-ab151739.html, PMID: 30015619<br>anti-DCAF8 Bethyl Laboratories® Catalog # A301-556A 1 for IF and WB, https://www.thermofisher.com/antibody/product/WDR42A-Antibody-Polyclonal/A301-556A<br>anti-myosin heavy chain DSHB Hybridoma Product MF 20, https://dshb.biology.uiowa.edu/MF-20<br>anti-GAPDH abcam: ab9485, https://www.abcam.com/products/primary-antibodies/gapdh-antibody-loading-control-ab9485.html<br>anti-HA-Tag (C29F4) Rabbit mAb cell signaling, HA-Tag (C29F4) Rabbit mAb #3724 1:1000 for WB<br>anti-CTCF Abcam, ab70303 (https://www.abcam.com/ctcf-antibody-ab70303.html).<br>anti-CTCF Millipore 07-729 https://www.emdmillipore.com/US/en/product/Anti-CTCF-Antibody,MM_NF-07-729?ReferrerURL=https%3A%2F%2Fwww.google.com%2F<br>anti-Rad21 Abcam, ab992, (https://www.abcam.com/rad21-antibody-ab992.html)<br>MyoD Santa cruz, Anti-MyoD Antibody (5.8A), sc-32758 X, https://www.scbt.com/p/myod-antibody-5-8a<br>anti-YY1 santa cruz, sc-7341X, https://www.scbt.com/p/yy1-antibody-h-10<br>Histone H3K4me3 antibody (pAb) active motif, 39159, https://www.activemotif.com/catalog/details/39159/histone-h3-trimethyl-lys4-antibody-pab<br>Anti-acetyl-Histone H3 (Lys27) Antibody, clone RM172 millipore, MABE647, https://www.emdmillipore.com/US/en/product/Anti-acetyl-Histone-H3-Lys27-Antibody-clone-RM172,MM_NF-MABE647

Eukaryotic cell lines

Policy information about cell lines and Sex and Gender in Research

Cell line source(s)	C2C12 cells were purchased from ATCC.
Authentication	Cell lines were not authenticated.
Mycoplasma contamination	All cell lines were tested negative for mycoplasma contamination by PCR.
Commonly misidentified lines (See ICLAC register)	None

Plants

Seed stocks

Report on the source of all seed stocks or other plant material used. If applicable, state the seed stock centre and catalogue number. If plant specimens were collected from the field, describe the collection location, date and sampling procedures.

Novel plant genotypes

Describe the methods by which all novel plant genotypes were produced. This includes those generated by transgenic approaches, gene editing, chemical/radiation-based mutagenesis and hybridization. For transgenic lines, describe the transformation method, the number of independent lines analyzed and the generation upon which experiments were performed. For gene-edited lines, describe the editor used, the endogenous sequence targeted for editing, the targeting guide RNA sequence (if applicable) and how the editor was applied.

Authentication

Describe any authentication procedures for each seed stock used or novel genotype generated. Describe any experiments used to assess the effect of a mutation and, where applicable, how potential secondary effects (e.g. second site T-DNA insertions, mosiacism, off-target gene editing) were examined.

ChIP-seq

Data deposition

☒ Confirm that both raw and final processed data have been deposited in a public database such as GEO.

☒ Confirm that you have deposited or provided access to graph files (e.g. BED files) for the called peaks.

Data access links
May remain private before publication.

For "Initial submission" or "Revised version" documents, provide reviewer access links. For your "Final submission" document, provide a link to the deposited data.

Files in database submission

Provide a list of all files available in the database submission.

Genome browser session
(e.g. UCSC)

Provide a link to an anonymized genome browser session for "Initial submission" and "Revised version" documents only, to enable peer review. Write "no longer applicable" for "Final submission" documents.

Methodology

Replicates

In general, ChIP-seq experiments were performed in at least two replicates. The number of replicates is indicated in each file name.

Sequencing depth

Describe the sequencing depth for each experiment, providing the total number of reads, uniquely mapped reads, length of reads and whether they were paired- or single-end.

Antibodies

anti-CTCF Abcam, ab70303
anti-Rad21 Abcam, ab992

Peak calling parameters

Specify the command line program and parameters used for read mapping and peak calling, including the ChIP, control and index files used.

Data quality

verified by FastQC

Software

Describe the software used to collect and analyze the ChIP-seq data. For custom code that has been deposited into a community repository, provide accession details.

Flow Cytometry

Plots

Confirm that:

☒ The axis labels state the marker and fluorochrome used (e.g. CD4-FITC).

☒ The axis scales are clearly visible. Include numbers along axes only for bottom left plot of group (a 'group' is an analysis of identical markers).

☒ All plots are contour plots with outliers or pseudocolor plots.

☒ A numerical value for number of cells or percentage (with statistics) is provided.

Methodology

Sample preparation

C2C12 cells were tripsinized and washed with growth medium. Cells were filtered and resuspended in 1X PBS with 1%FCS and 7-AAD.

Instrument

JF Aria II

Software

FlowJo

Cell population abundance	select the top 0.2% GFP positive cells
Gating strategy	a figure exemplifying the gating strategy is provided in the Supplementary Information

☒ Tick this box to confirm that a figure exemplifying the gating strategy is provided in the Supplementary Information.
