# Supplementary material for: Matrin3 mediates differentiation through stabilizing chromatin loop-domain interactions and YY1 mediated enhancer-promoter interactions
Source: Nat Commun. 2024 Feb 10;15:1274. doi: 10.1038/s41467-024-45386-w (PMC10858947; doi:10.1038/s41467-024-45386-w)

Figure 1b

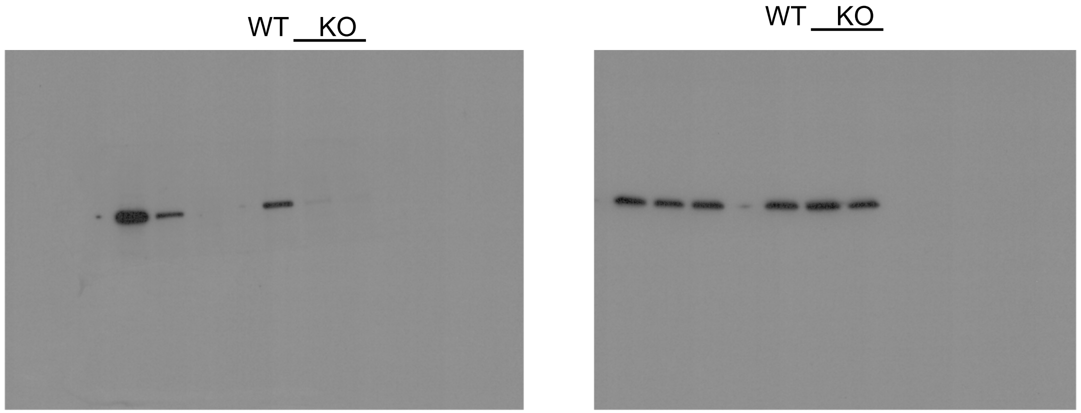

Antibody: Matr3 Antibody: GAPDH

Figure 1e

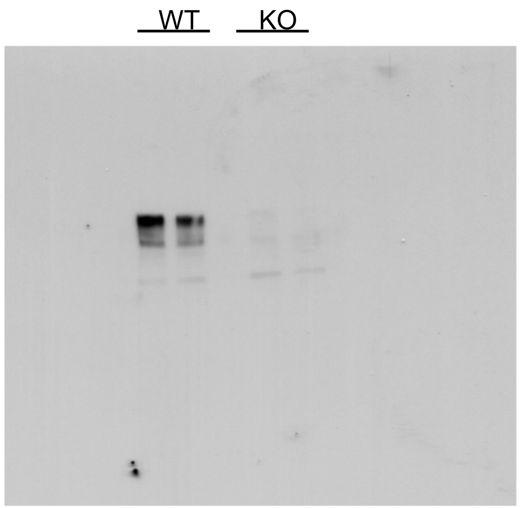

Antibody: DMD

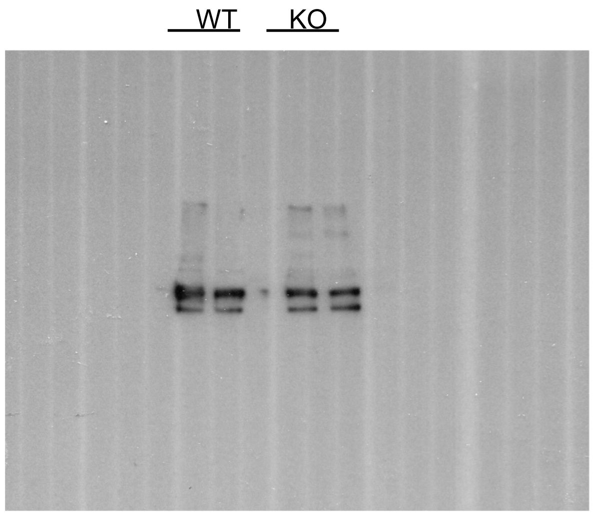

Antibody: MHC

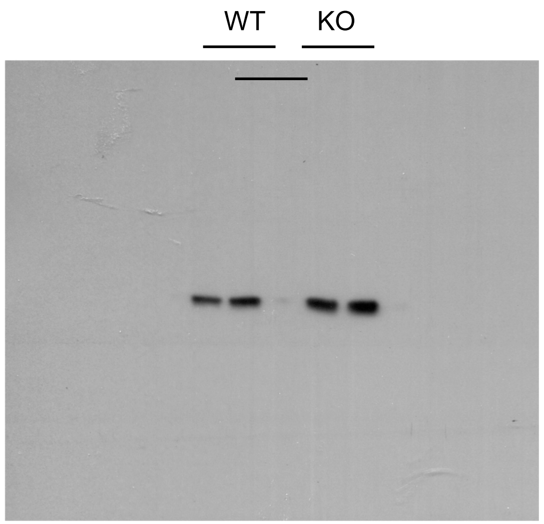

Antibody: GAPDH

Figure 1f

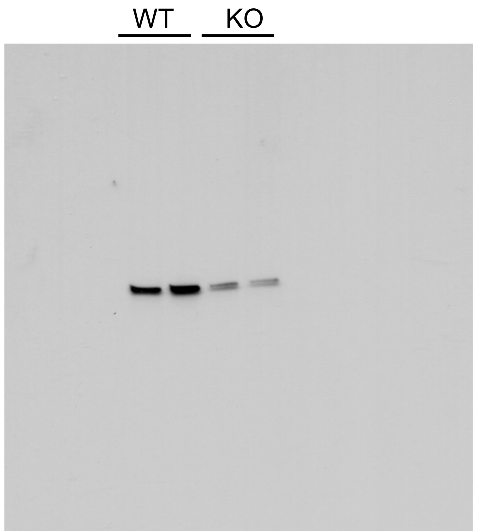

Antibody: DCAF8

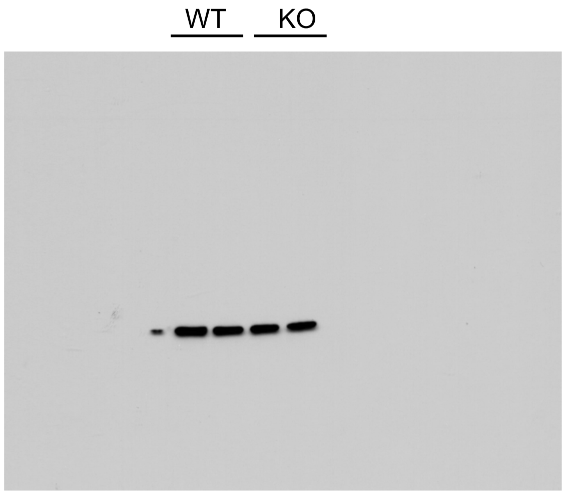

Antibody: GAPDH

Figure 2b

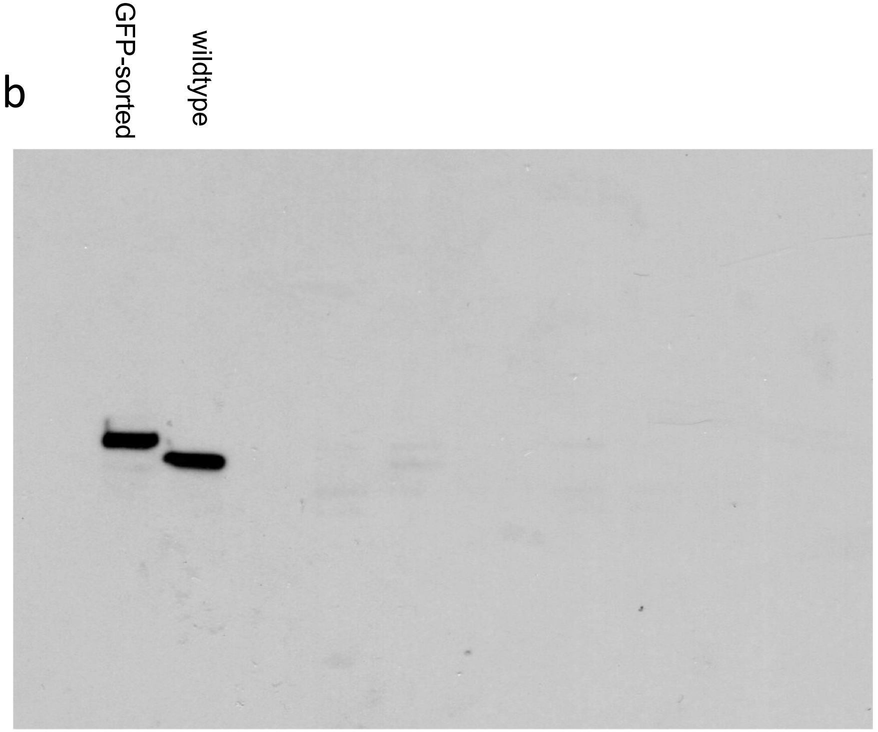

Antibody: MATR3

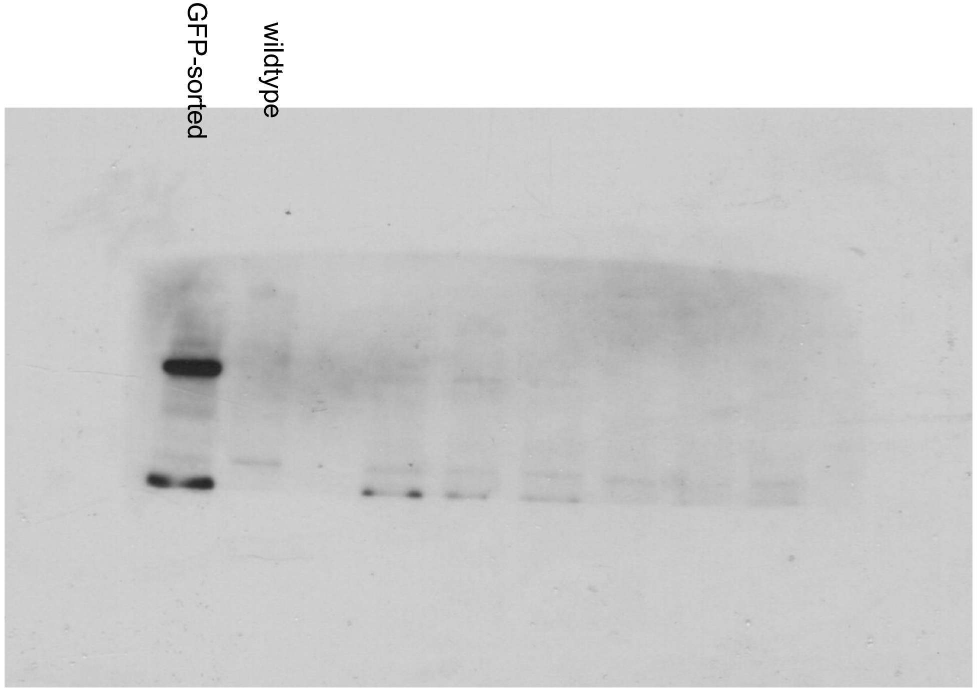

Antibody: HA

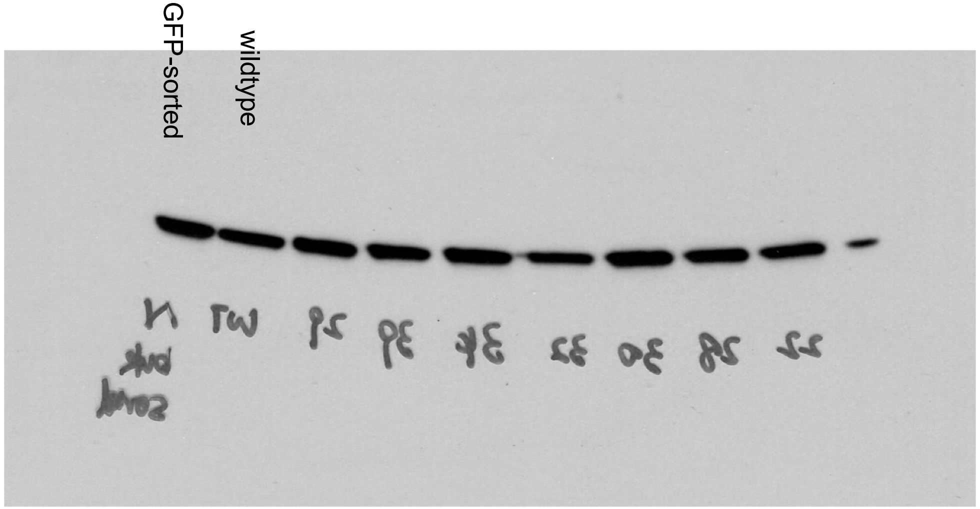

Antibody: GAPDH

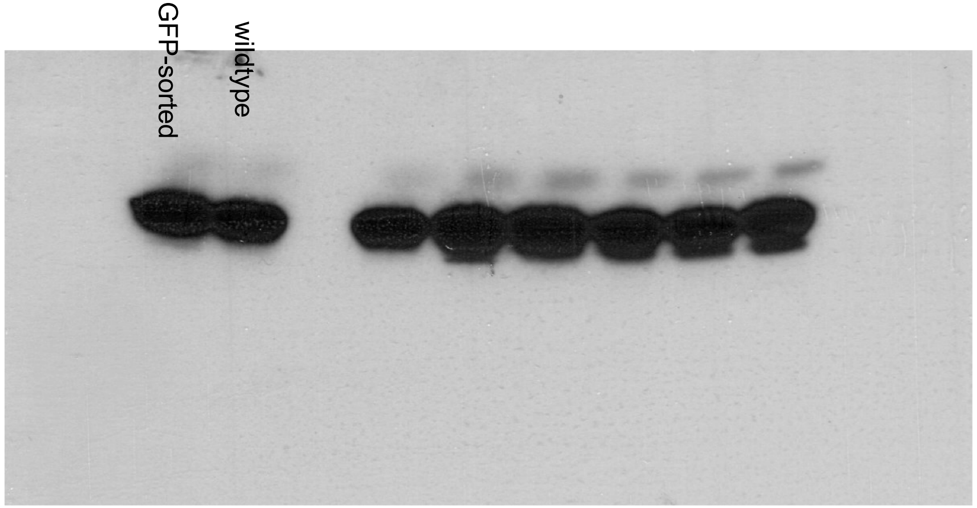

Antibody: GAPDH

Figure 2c

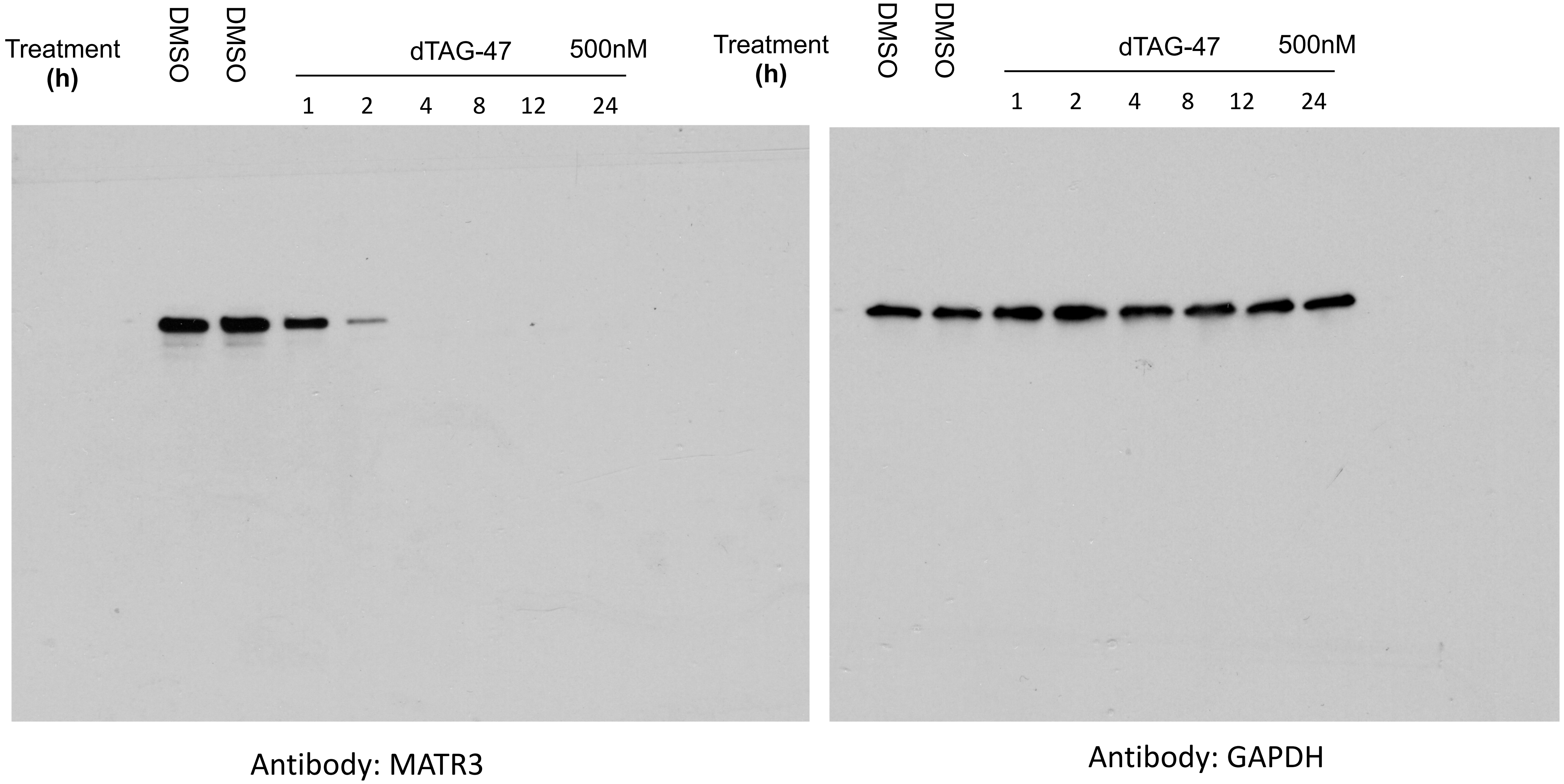
Treatment (h)
DMSO
DMSO
dTAG-47 500nM
1
2
4
8
12
24
Antibody: MATR3
Treatment (h)
DMSO
DMSO
dTAG-47 500nM
1
2
4
8
12
24
Antibody: GAPDH

Supplementary Figure 1b

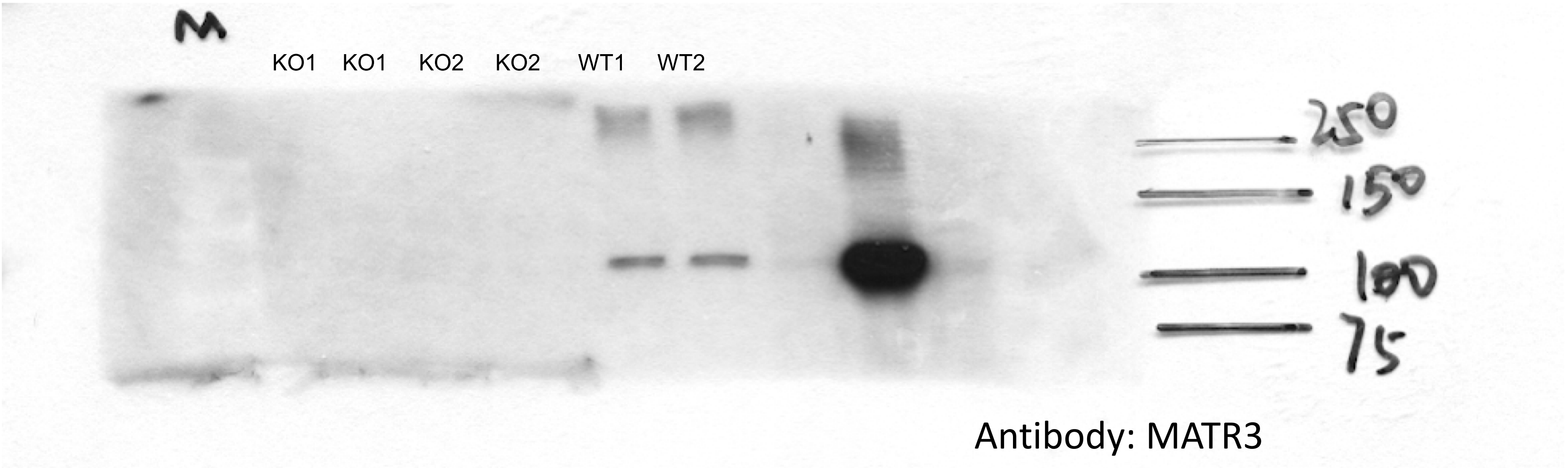

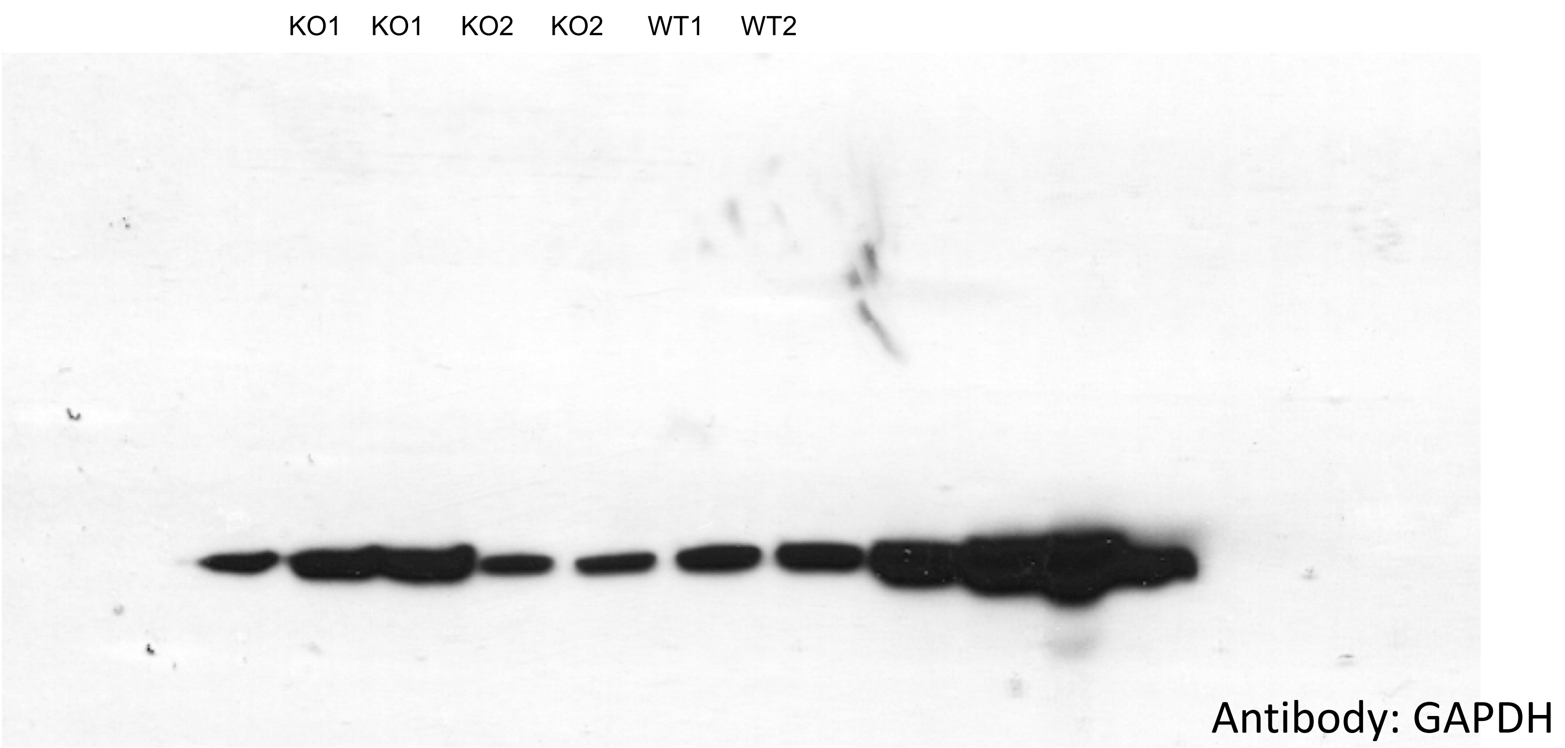

Supplementary Figure 4a

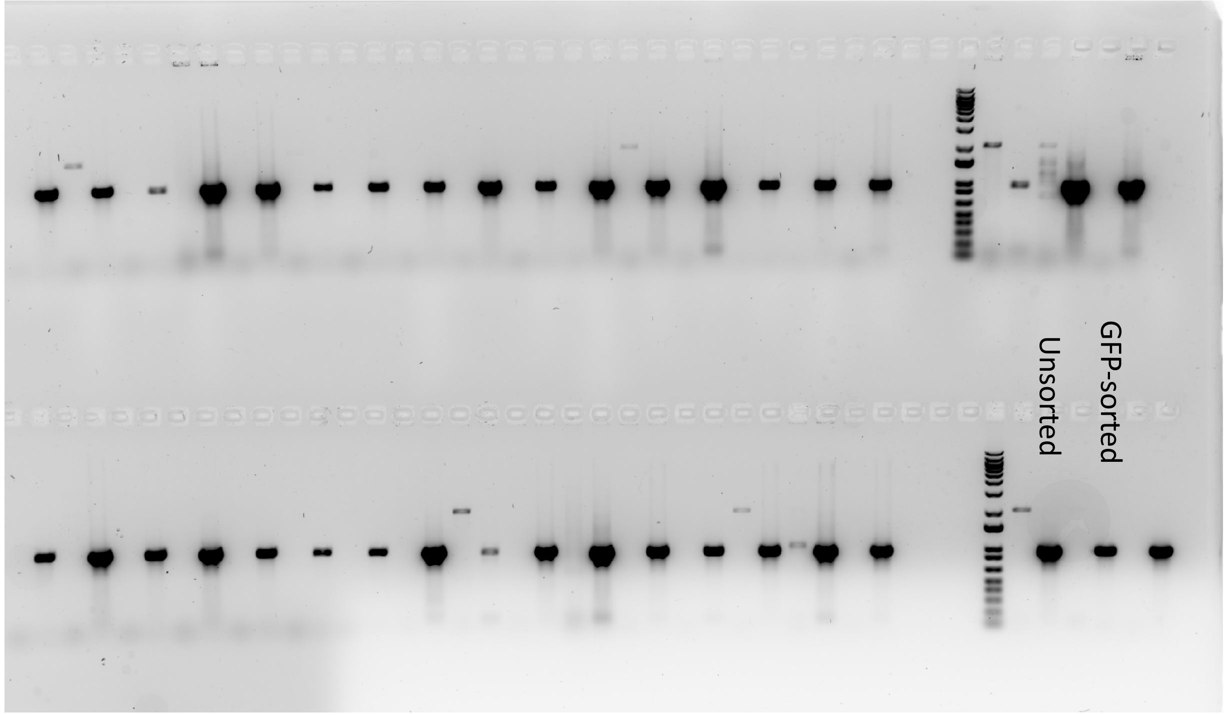

DNA Agarose gel

Supplementary Figure 4b

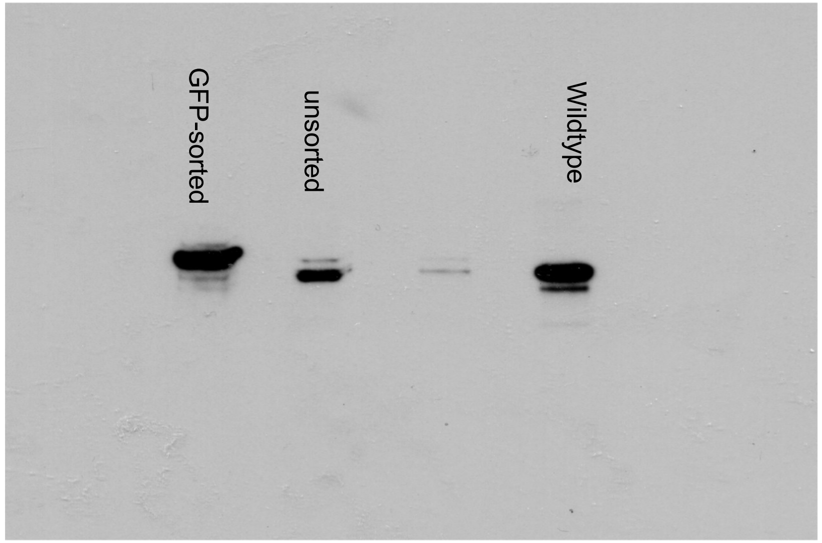

Antibody: MATR3

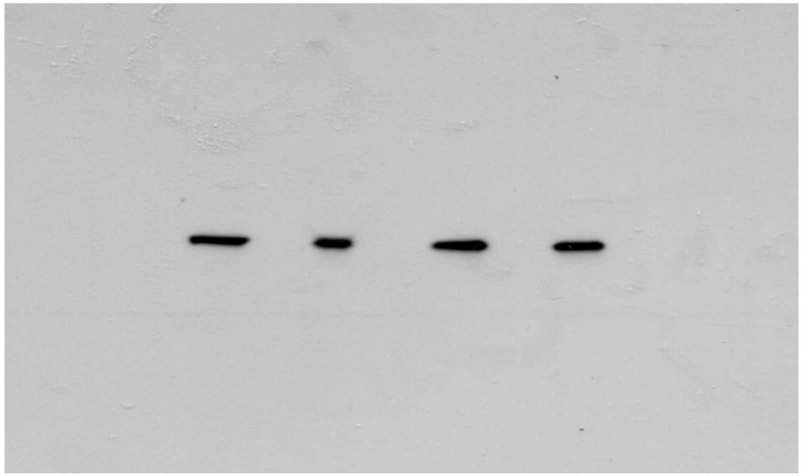

Antibody: GAPDH

Supplementary Figure 4c

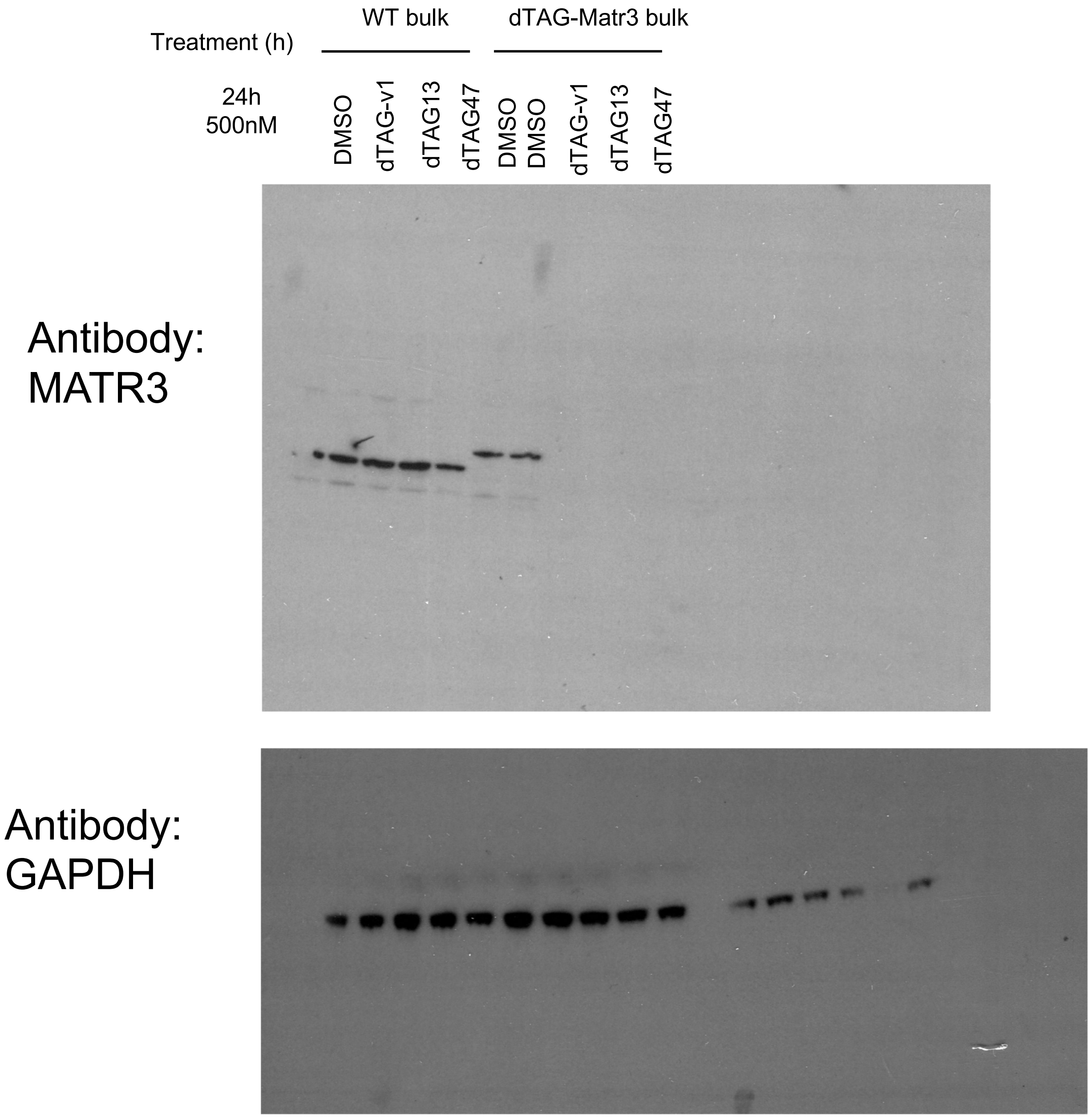

Supplementary Figure 4d

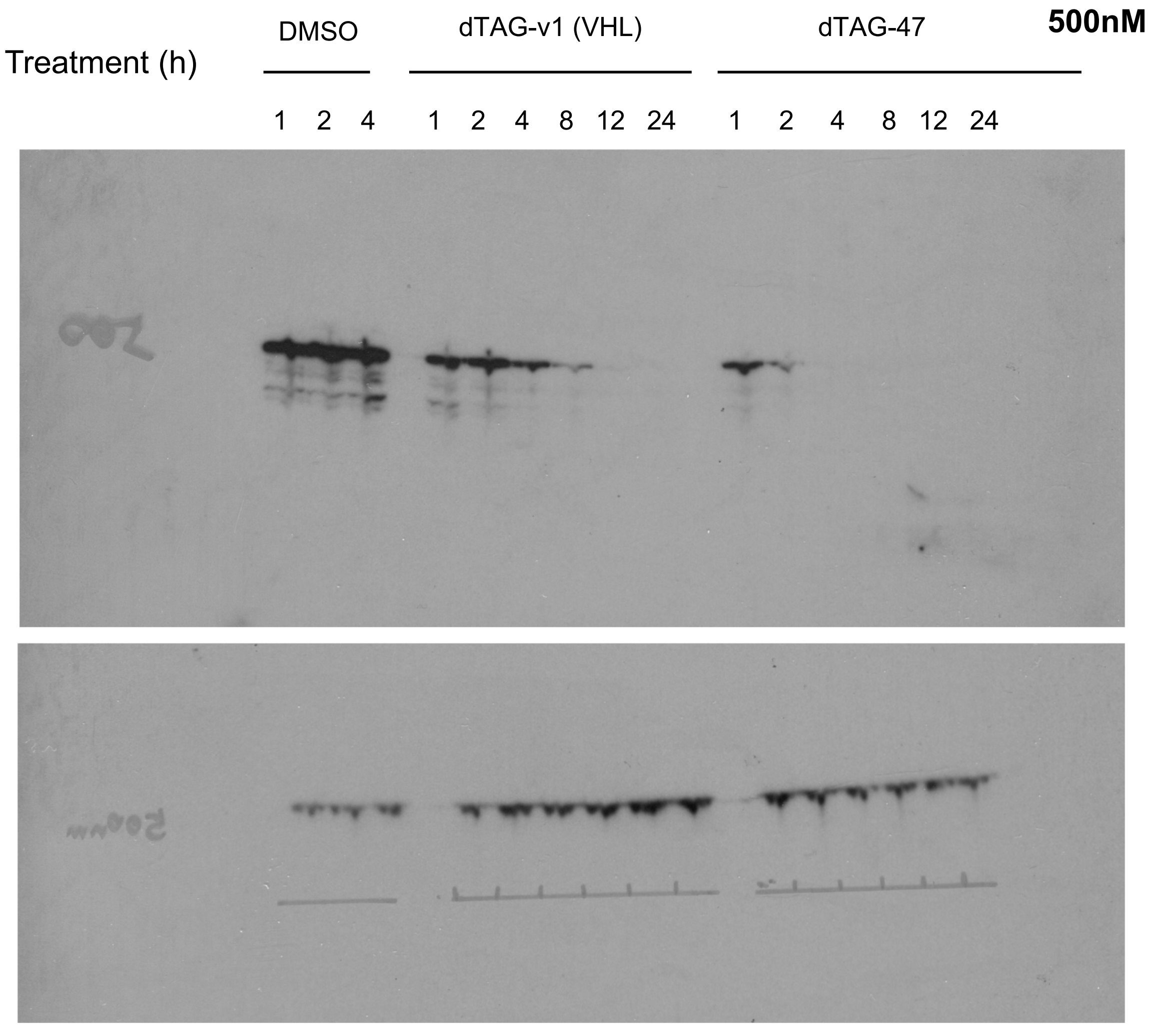

Supplementary Figure 5a

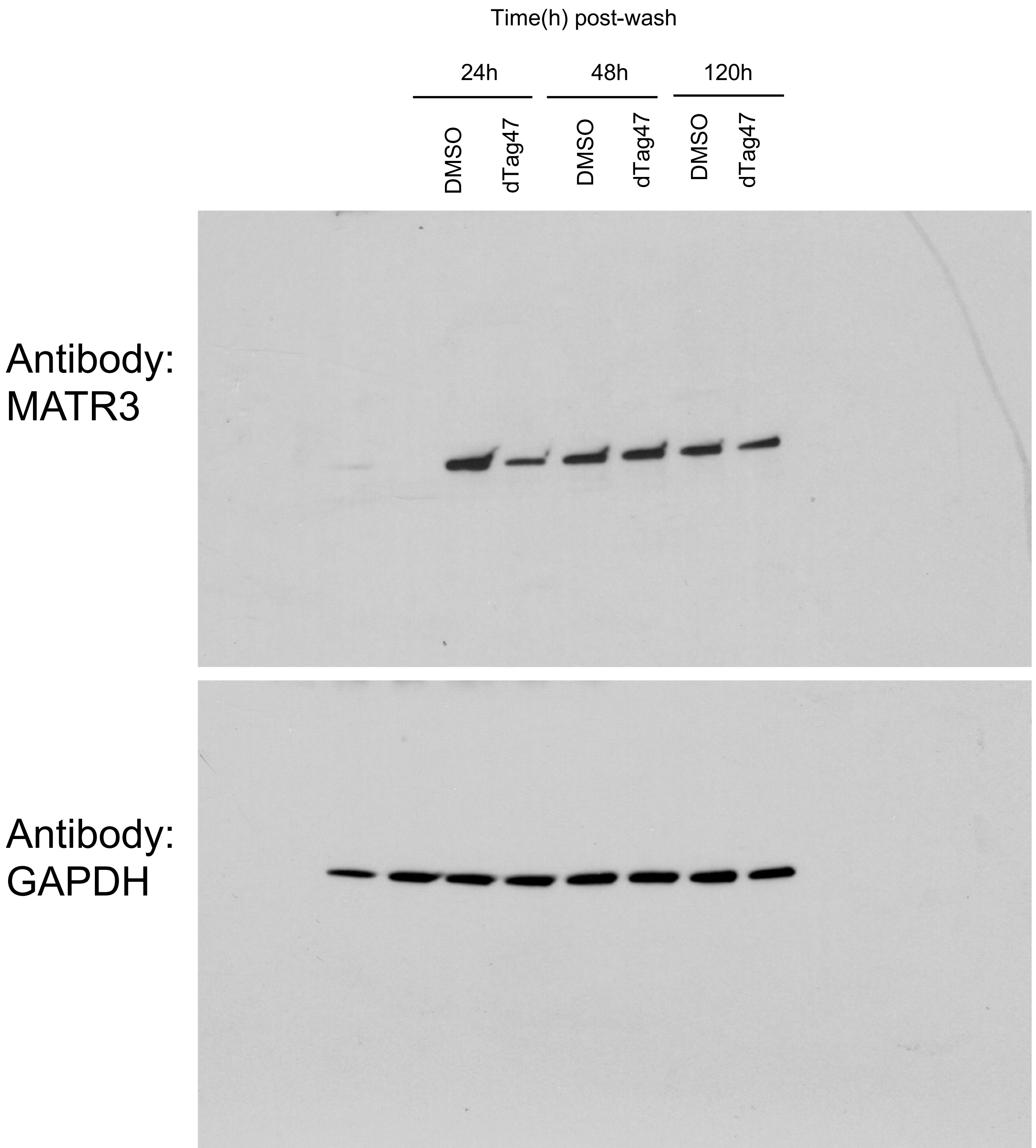

Supplementary Figure 10a

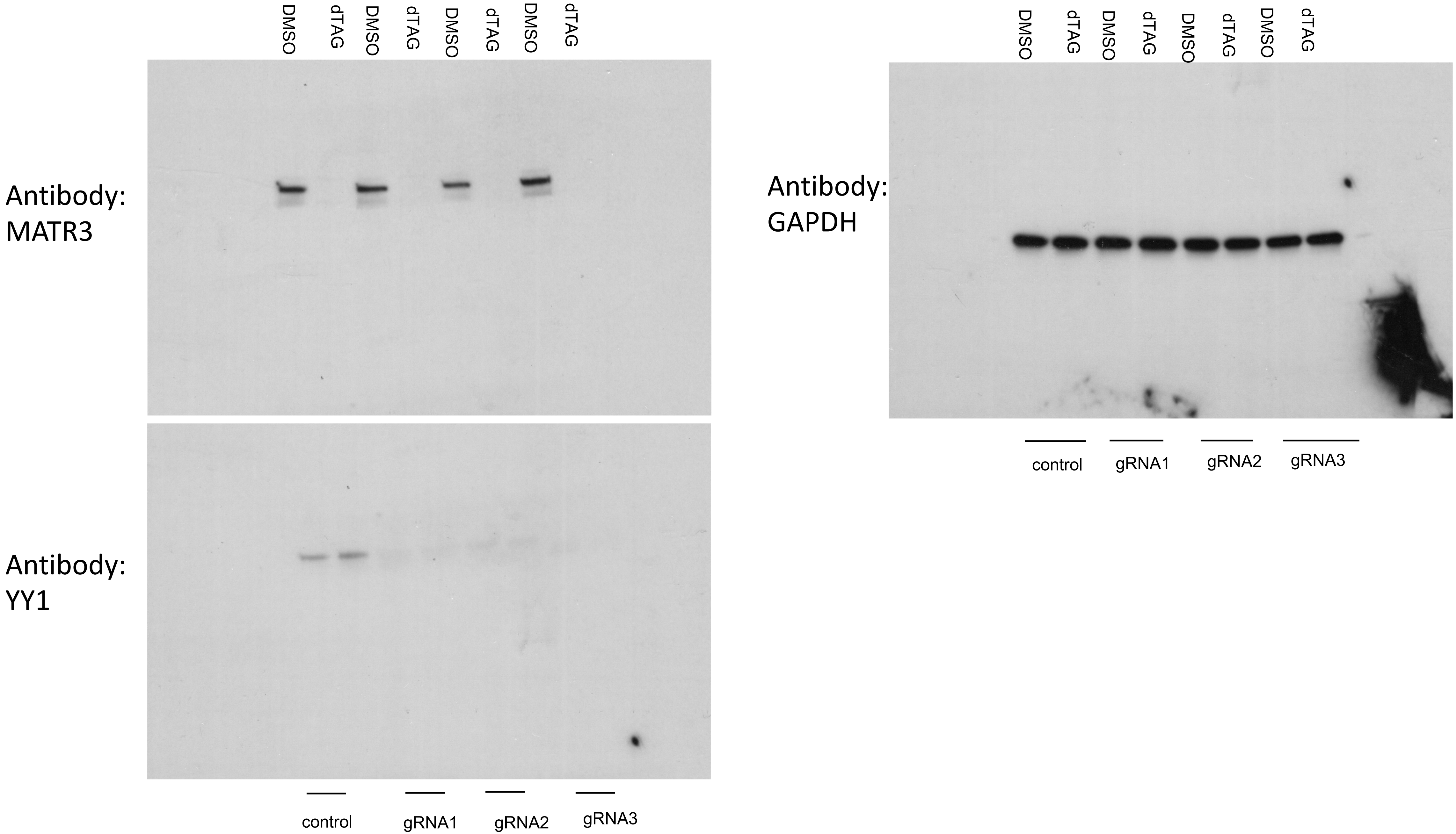

Supplement: Supplementary file 4 — Source Data [file 41467_2024_45386_MOESM4_ESM.zip › Souce data 12202023/Source data.pdf]
